# Improvement of Polyunsaturated Fatty Acid Production in *Echium acanthocarpum* Transformed Hairy Root Cultures by Application of Different Abiotic Stress Conditions

**DOI:** 10.5402/2013/169510

**Published:** 2013-11-13

**Authors:** Rafael Zárate, Elena Cequier-Sánchez, Covadonga Rodríguez, Roberto Dorta-Guerra, Nabil El Jaber-Vazdekis, Ángel G. Ravelo

**Affiliations:** ^1^Canary Islands Cancer Research Institute (ICIC), 61 Avenida La Trinidad, Torre A. Arévalo, 7th Floor, 38204 La Laguna, Tenerife, Spain; ^2^Bio-Organic University Institute A.G. González, University of La Laguna, Ave. Fco. Sánchez, 38206 La Laguna, Tenerife, Spain; ^3^Animal Biology Deptartment (Physiology Unit), Biology Faculty, University of La Laguna, Ave. Fco. Sánchez, 38206 La Laguna, Tenerife, Spain; ^4^Institute of Biomedical Technologies (ITB), University of La Laguna, Campus de Ofra, 38071 La Laguna, Tenerife, Spain; ^5^Statistics and Computation Deptartment, Maths Faculty, University of La Laguna, Ave. Fco. Sánchez, 38206 La Laguna, Tenerife, Spain

## Abstract

Fatty acids are of great nutritional, therapeutic, and physiological importance, especially the polyunsaturated n-3 fatty acids, possessing larger carbon chains and abundant double bonds or their immediate precursors. A few higher plant species are able to accumulate these compounds, like those belonging to the *Echium* genus. Here, the novel *E. acanthocarpum* hairy root system, which is able to accumulate many fatty acids, including stearidonic and *α*-linolenic acids, was optimized for a better production. The application of abiotic stress resulted in larger yields of stearidonic and *α*-linolenic acids, 60 and 35%, respectively, with a decrease in linoleic acid, when grown in a nutrient medium consisting of B5 basal salts, sucrose or glucose, and, more importantly, at a temperature of 15°C. The application of osmotic stress employing sorbitol showed no positive influence on the fatty acid yields; furthermore, the combination of a lower culture temperature and glucose did not show a cumulative boosting effect on the yield, although this carbon source was similarly attractive. The abiotic stress also influenced the lipid profile of the cultures, significantly increasing the phosphatidylglycerol fraction but not the total lipid neither their biomass, proving the appropriateness of applying various abiotic stress in this culture to achieve larger yields.

## 1. Introduction

Lipids in general and fatty acids (FA) in particular are essential metabolites displaying many key biological functions, acting as structural components of cell membranes, energy sources, and known intermediates in signaling pathways, besides their broad interest due to their important roles in human health and nutrition [1–6].

Oil producing plants could be an alternative dietary ingredient source of omega or n-3 polyunsaturated fatty acids (PUFA) for the aquaculture industry; thus, it would be interesting to establish how the environmental factors modulate PUFA production in plants. These lack the ability to move to avoid possible environmental stress situations and, therefore, have to adapt to their environment in many different ways, and, similar to that described for fishes, temperature is one of the most influential environmental factors. Cold acclimation and the acquisition of freeze tolerance require the orchestration of many different seemingly disparate physiological and biochemical changes, including increasing sugar levels, soluble proteins, proline, certain organic acids, and new protein isoforms, alteration of lipidic membrane, and particularly differential expression of many genes coding for effector molecules that participate directly to alleviate stress [7]. 

Biological membranes are considered liquid crystals that behave as two-dimensional fluids. Thus, FA are able to participate in different adaptation mechanisms of plants to stress conditions, particularly through modification of the cell membrane fluidity and permeability. Furthermore, unsaturated FA facilitate the fluidity of lipids, having fewer van der Waals interactions, with the position of the double bonds in FA being more influential than their number [10]. Therefore, the addition of one or two double bonds to the membrane FA drastically decreases the transition temperature, and apparently the addition of a third or fourth double bond does not affect it. However, trienoic FA (e.g., 16:3 or 18:3) are the most abundant in chloroplast membranes, and increasing them allows a larger plant tolerance against low temperatures [11]. Inversely, in transgenic tobacco plants, in which the gene encoding chloroplast omega-3 fatty acid desaturase, which synthesizes trienoic FA, was silenced, displayed a lower level of trienoic FA than wild-type plants, offering a better acclimation to higher temperatures [12].

The PUFA dependence of membrane fluidity as well as of other physical properties including those necessary for membrane fusion events is a well-characterized phenomenon in animals including fish, fungi, bacteria, and plants [13–17], together with the changes that occur when temperature increases or decreases. Unsaturated FA are thought to aid in maintaining membranes in a fluid state necessary for an appropriate biological functioning [18, 19].

In plants, FA such as 16:0 and 18:1n-9 are used to form lipid membranes by means of two different metabolic pathways: the chloroplast pathway or the cytoplasmic pathway, occurring in the endoplasmic reticulum (ER). In the first one, the FA of 16 carbons is generally esterified in the second position (sn-2) of the glycerol backbone, while in the ER within the synthesized lipids predominate those with 18 carbons. From here on, the desaturation process continues catalyzed by different enzymes which add an unsaturation to 18:1n-9, resulting in 18:2n-6, and so forth. Although there are differences between the desaturases found in chloroplasts (FAD7, FAD8) and those found in the ER (FAD2, FAD3) or in nonphotosynthetic tissues. The expression of each of their coding genes and enzymes is regulated by a complex system involving a variety of environmental stimuli, such as light or temperature, stress (damage, salt stress, pathogen invasion, etc.), or in response to the presence of jasmonate in the medium. Transgenic plants expressing or silencing enzymes of the biosynthetic pathway of PUFA have allowed the artificial modification of membrane FA and their physical and physiological adaptation to low temperatures. For instance, in *Arabidopsis* mutants deficient in one or more desaturase enzymes (*fad* genes), the degree of FA unsaturation was extremely important in the plant response to cold temperatures [20], in particular in chloroplasts, where trienoic FA are important to ensure the correct biogenesis and maintenance of chloroplasts during plant growth at low temperatures [21]. 

Several authors have suggested that temperature is a factor capable of regulating even the expression of desaturase enzymes in several ways, such as transcription, posttranscription, and translational. In soybean seeds, low temperatures were applied and the data showed how D12 and D15 desaturases could be rapidly modulated in response to altered growth temperatures, while the enzymes for FA synthesis and elongation were not [22]. The regulation of these enzymes by temperature was documented with *Brassica napus fad3* desaturase gene expressed in yeast; an increase in 18:3n-3 and FAD3 enzyme was recorded at low temperatures, but no *fad3* transcript was observed, suggesting that a posttranscriptional regulation was taking place [23].

Regarding the influence of osmotic stress on the FA profiles, osmotic pressure plays a crucial role as a regulator in the cellular water balance. Despite this important role, in *in vitro* plant culture systems employed for the production of secondary metabolites, much more attention has been applied to other factors, such as temperature or the nutrients present in the culture medium [24]. Few studies have addressed the use of osmotic stress or high osmotic pressure to stimulate the production of secondary metabolites in plant cell cultures. The application of osmotic stress on cells suspension cultures of *Catharanthus roseus* resulted in an increase in the intracellular accumulation of catharanthine and other alkaloids [25]. Other studies showed the influence of osmotic stress on anthocyanin production in cell culture of *Vitis vinifera* [26], in *Populus deltoides* [27], and in cell cultures of *Panax notoginseng* [28], and, more recently, it was applied in cell cultures of *C. roseus* [29], *Taxus chinensis* [30], and *Panax ginseng* [31], in order to induce the production of various alkaloids, paclitaxel, and saponins, respectively. In these studies, it has been confirmed that although occasionally the addition of sorbitol or mannitol can increase the production of metabolites of interest, it may also decrease the fresh weight of the cultures. Regarding the relationship between the induced stress and the production of PUFA, some studies have suggested that the hyperosmotic stress may reduce membrane fluidity similar to low temperatures. Contrary, the hypoosmotic stress effect is not well documented, but it has also been suggested that as the temperature is high, hypoosmotic stress may increase membrane fluidity.

Analogously, in the aquatic environment in general and in the field of fish aquaculture in particular, it is important to take into account environmental factors, especially temperature, a factor that directly influences the development and yield of crops. Cold affects fish growth and health and may decrease fish-farm production, even causing mortality through what is known as “winter syndrome” [32]. In some species adapted to very low temperatures, there is an interesting effect in order to keep the fluidity of cell membranes; they can decrease the chain length or increase the number of double bonds of those FA esterified in phospholipids [33]. Moreover, in deep-see living fish, it has been shown that high hydrostatic pressures exert the same influence as low temperatures, showing changes in certain enzyme activities, an increase in oxygen consumption, and increased membrane fluidity, achieved by increasing the unsaturation of FA esterified with phospholipids [33, 34]. 

In this study, we report the different strategies carried out in a novel *Echium acanthocarpum* transformed hairy root system, in order to particularly increase the production of the unusual PUFA, stearidonic (SDA; 18:4n-3) and *γ*-linolenic acids (GLA; 18:3n-6), of increasing pharmacological interest, by reducing culture temperature, applying osmotic stress, and changing the carbon source. Moreover, in order to determine the validation of the data and the effectiveness of the abiotic stress, a robust statistical approach was applied.

## 2. Materials and Methods

### 2.1. Plant Material

Seeds of *E. acanthocarpum*, donated by Jardín Botánico Viera y Clavijo, Gran Canaria, Spain, were first surface-sterilized by a brief immersion in 70% EtOH, followed by submersion in an aqueous solution of 5% (v/v) of commercial bleach for 25 min with gentle hand agitation. They were finally washed 5 times with sterile distilled water.

Treated seeds were then germinated *in vitro* on a solid B5 [8] medium, supplemented with 3% sucrose, 3-4 mg/L GA3 (gibbereelic acid), and solidified with 0.7% agar, with the pH adjusted to 6.0 prior to autoclaving (115°C, 1 atm. pressure, 15 min.), contained in Petri dishes (90 mm diameter), and cultured in the dark until the beginning of germination. After germination, plants were transferred to the same solid nutrient medium without the addition of GA3, contained in translucent glass jars covered with a lid (175 mL capacity, Sigma-Aldrich, MO, US), which were placed under light conditions (16 h photoperiod and irradiance of 35 mmol m^2^s^−1^ supplied by cool white fluorescent tubes) and a temperature of 25 ± 2°C to allow further plant growth.

Under sterile conditions, 50–60-day-old plants were employed for guided infection with *Agrobacterium rhizogenes* strain LBA1334 harboring a pBIN19-gus intron plasmid by repeatedly stabbing the internodal stem areas with a fine needle containing bacteria [35, 36]. After 25–30 days, hairy roots of 3-4 mm in length had developed and were aseptically excised and transferred to B5 liquid medium, containing the antibiotic cefotaxime (100 mg/L) as well as 1% of the antioxidant polyvinylpyrrolidone (PVP) for several subcultures. Finally, actively growing bacterium-free hairy roots were cut into small segments and routinely cultured and refreshed in Erlenmeyer flasks (250 mL), containing 30 mL of sterile B5 liquid medium supplemented with 3% sucrose and 1% of PVP (standard nutrient medium), sealed with a double layer of aluminum foil, and placed on an orbital shaker at 95 rpm at 25 ± 2°C in the dark.

For culture growth and FA production and analysis, different hairy root culture media of the established E1.5 cell line were investigated, each providing a particular abiotic stress and culture conditions (Table 1). In order to cover the entire growth period for each culture, sampling times were different since the kinetics of growth differed due mainly to the culture temperature; thus, the sampling points were as follows T1, 5 days for culture B1 and 15 days for cultures C1–C4; T2, 10 days for culture B1 and 25 days for cultures C1–C4; T3, 15 days for culture B1 and 35 days for cultures C1–C4; T4, 20 days for culture B1 and 45 days for cultures C1–C4; T5, 25 days for culture B1 and 65 days for cultures C1–C4; and T6, 35 days for culture B1 and 75 days for cultures C1–C4.

### 2.2. Lipid Extraction and Transesterification of Lipids

Hairy roots were separated from the liquid nutrient medium by vacuum filtration, weighed, and lyophilised at −80°C for 24 h using a freeze-dryer (Christ Alpha 2-4, Osterode, Germany). Each sample was powdered using a mortar and pestle with liquid nitrogen. After homogenisation, total lipid was extracted following the method previously described [36–38].

Lipid aliquots (2 mg) were subjected to acid-catalyzed transesterification by dissolving the sample in 1 mL toluene, employed to ensure that the neutral lipids got properly dissolved, plus 2 mL of a mixture of MeOH/1% H_2_SO_4_, and incubated in a capped glass test tube at 50°C for 16 h [39]. Prior to transmethylation, heneicosae-noic acid (21:0) (2.5% of the total lipid analysed, 50 *μ*g), was added as internal standard to the lipid extracts. Transesterification was conducted as previously described [36–38]. Preparative thin layer chromatography employing silica gel G-25 glass sheets (Macherey-Nagel, Germany), developed with a solvent system composed of hexane/diethyl ether/acetic acid 97.7% (90 : 10 : 1, by vol) and visualized after brief sublimation of iodine with slight heat, was used for the isolation and purification of the fatty acids methyl esters (FAMEs). These ran close to the solvent front and were then scrapped off the glass sheet, extracted with 10 mL hexane/ethyl ether (1 : 1, v/v), and dried under nitrogen. Finally, the samples were dissolved in 0.5–1.0 mL hexane and kept under nitrogen in sealed glass vials at −20°C until analysis.

### 2.3. Gas Chromatography of FAMEs

Analysis and quantification of FAMEs were conducted by GC employing a Shimadzu GC-14A apparatus (Shimadzu, Japan) equipped with a flame ionization detector (250°C), a Supelcowax 10 fused silica capillary column (30 m × 0.32 mm ID), employing helium as carrier gas. Samples (0.6 *μ*L) were injected into the system by an on-column autoinjector (Shimadzu AOC-17) at 50°C. A temperature program of 180°C for the first 10 min, followed by an increase of 2.5°C/min until reaching 215°C, was employed for separation of the compounds.

FAMEs were identified according to their RT compared with standards of individual commercial FAMEs (linoleic acid methyl ester, methyl *γ*-linolenate, methyl oleate, stearidonic acid methyl ester, and heneicosanoic acid) and a well-characterized fish oil mix (Sigma-ref LUPE). They were quantified according to the amount of 21:0 added as internal standard prior to transmethylation and by comparison with a calibration curve created with the individual standards.

### 2.4. Statistical Analysis

Results are present as the means and standard deviations of three replicates for each sampling time for each of the treatments and cultures. The data were checked for normal distribution by one-sample Kolmogorov-Smirnov test as well as for homogeneity of the variance with the Levene test, and, when necessary, Bartlett test was also applied. When variance was not homogeneous, Kruskal-Wallis and Games-Howele tests were conducted to assess statistical differences. The effects of culture conditions and FA levels were firstly determined using one-way ANOVA test (*P* < 0.05). The percentages and total amounts of FA, particularly the contents of GLA and SDA in the different cultures, were included as variables in a principal component analysis (PCA). Principal components were subsequently analysed by two-way ANOVA to study the combined effects of both factors, FA profiles and stress conditions, as well as their interconnections. Statistical analyses were performed employing the SPSS software (versions 15.0 and 17.0, SPSS Inc., IL, USA).

## 3. Results and Discussion

Studies of growth conditions and type of stress applied to cultures were conducted in order to achieve FA profiles richer in Δ6-desaturated products, such as SDA and GLA. 

### 3.1. Effect of Stress on *Echium acanthocarpum* Hairy Roots Growth

In order to carry out these experiments, hairy roots from line E1.5 were used taking into account the previously described results [36, 40]. The data of growth were recorded under different nutrient media and conditions (cultures B1, C1, C2, C3, and C4). 

**Figure 1:**
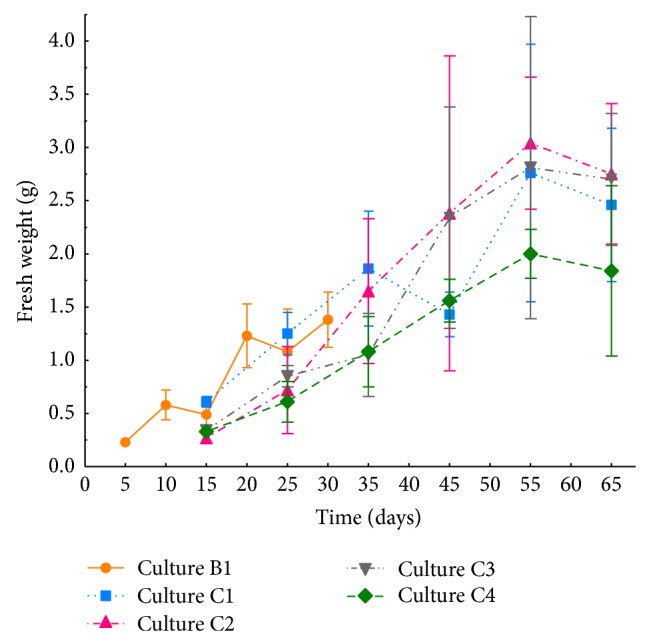
Fresh weight (g) variation of *Echium acanthocarpum* transformed hairy roots cell line E1.5 cultured in three different growth media and temperatures. Values represent the mean of three replicates (*n* = 3)  ±  SD. Depending on the culture temperature 25°C (culture B1) or 15°C (cultures C1–C4), the sampling points varied as *T*1 = 5 days for culture B1 and 15 days for cultures C1–C4; *T*2 = 10 days for culture B1 and 25 days for cultures C1–C4; *T*3 = 15 days for culture B1 and 35 days for cultures C1–C4; *T*4 = 20 days for culture B1 and 45 days for cultures C1–C4; *T*5 = 25 days for culture B1 and 55 days for cultures C1–C4; and *T*6 = 30 days for culture B1 and 65 days for cultures C1–C4.

A typical growth curve was achieved for all cultures, with a lag phase, which tended to be more pronounced in cultures C2–C4 than culture C1, at sampling points 1 and 2 (Figure 1). Then, the cultures started their exponential growth phase. Finally, the cultures described a stationary phase (points 4 and 5), and from that point on, cell death took place, in which cultures lost their hairy root morphology, showing also vitrification and browning.

In culture B1, a maximum fresh weight of 1.38 g was registered at sampling point 6, whereas the maximum of culture C1 was 2.76 g at sampling point 5. Statistical analysis of fresh weight of both cultures showed only significant difference for sampling points 1 and 2 (Figure 1).

In relation to the carbon source used, when glucose was used instead of sucrose, it would be expected that glucose would be able to induce a faster growth, particularly in the lag phase, since sucrose would require to be hydrolyzed into the monomers glucose and fructose to then be taken up by the tissues. However, both carbon sources displayed comparable growth rates (Figure 1). Thus, when fresh weight variation of hairy roots cultured at 15°C (cultures C1–C4) was compared, no significant differences were observed in any of the analyzed points, except for the first one (Figure 1). In culture C2, containing 0.2 M sorbitol as osmotic pressure inducing agent, fresh weight reached 3.04 g at sampling point 5. Similarly, in cultures C3 and C4, the maximum values were recorded at sampling point 5, 2.81 and 2.00 g, respectively, although no significant differences were observed in any of the sampling points (Figure 1). Interestingly, it has been described that the application of osmotic stress tends to decrease the fresh weight of *in vitro* cultured rice or wheat callus; this is mainly due to the accumulation of metabolites or osmolytes in the cytosol, such as glycinebetaine, proline, or soluble carbohydrates, which balance the effect of the osmotic pressure produced by the extracellular solute concentration, avoiding the massive loss of intracellular water [41–43]. This fact would not be present in *E. acanthocarpum* hairy roots, since no reduction in fresh weight was observed, but a rather slight increase. Taking into account the final fresh weight versus the initial fresh weight, culture C2 consisting of B5 mineral salts, 3% sucrose, 1% PVP and 0.2 M sorbitol was the most effective, with a 12-fold increase, whereas fresh weight of C1, C3, and C4 increased in the range of 8–11 folds. Nonetheless, a negative interaction of sorbitol on the growth of certain *in vitro* cultures, such as ginseng roots, with 3% sucrose was published. The data showed a considerable growth reduction when sorbitol (0.2–0.3 M) was added [44]. However, a beneficial growth effect upon the addition of sorbitol (54.97 mM) to a certain varieties of rice *in vitro* cultures has also been described [41]. It might be possible that sorbitol, a polyhydric alcohol sugar, could be metabolized by tissues when sucrose amounts get finished. In our study, the addition of sorbitol does not show any detrimental effect on growth but a rather slight nonsignificant increase.

### 3.2. Effect of Stress on *Echium acanthocarpum* Hairy Roots Lipid Composition: Principal Component Analyses

**Figure 2:**
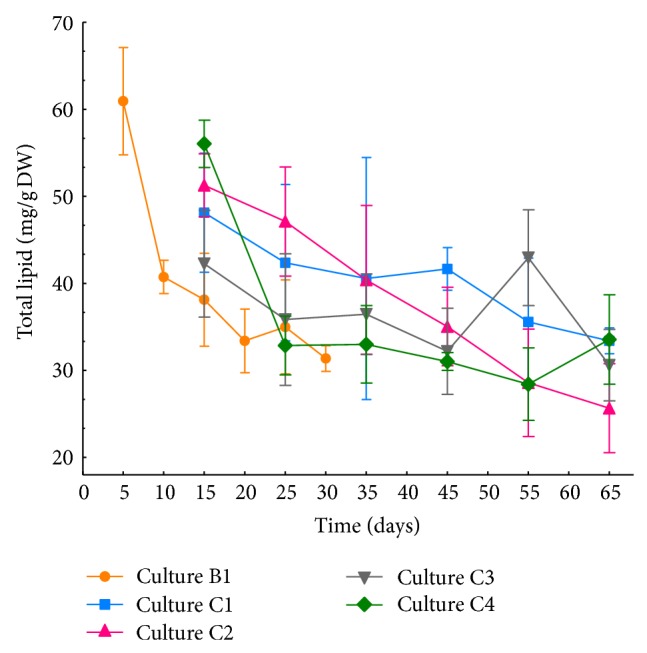
Total lipid (TL) content (mg/g DW) of E1.5 *Echium acanthocarpum* transformed hairy root cell line cultured in different culture media and temperatures. Values represent the mean of three independent replicates (*n* = 3) ± SD. Depending on the growth temperature, the sampling points varied as *T*1, 5 days for culture B1 and 15 days for cultures C1–C4; *T*2, 10 days for culture B1 and 25 days for cultures C1–C4; *T*3, 15 days for culture B1 and 35 days for cultures C1–C4; *T*4, 20 days for culture B1 and 45 days for cultures C1–C4; *T*5, 25 days for culture B1 and 65 days for cultures C1–C4; and *T*6, 35 days for culture B1 and 75 days for cultures C1–C4.

In general, the total lipid (TL) extracted from culture C1 hairy roots grown at 15°C was statistically similar to that of culture B1 grown at 25°C (Figure 2). The temperature did not significantly affect TL, and apparently *E. acanthocarpum *hairy roots are able to maintain homeostasis under these conditions. The data also indicate the possibility of further reducing the culture temperature to likely boosts its effect, on the production of desired Δ6-desaturated fatty acids, GLA, and SDA, as has been published for *B*. *napus* cultures grown at 4°C [45] or *Arabidopsis* grown at 12°C [46].

Analogously, the addition of sorbitol and/or glucose or both (cultures C2, C3, C4) did not seem to have a direct influence on TL since mostly no statistical differences were recorded except for sampling point 4 (Figure 2), although some reports showed that under water stress, total lipid clearly dropped in leaves of *A. thaliana* and *Cocos nucifera* [47, 48]. The decrease in TL is often associated with the richness of saturated FA in the leaf, as they are a more accessible substrate for hydrolysis and peroxidation reactions [49] and also because the leaves contain chiefly monogalactosyl diacylglycerides (MGDG) and digalactosyl diacylglycerids (DGDG), which are mainly esterified with saturated FA under stress conditions. Therefore, some authors claim that chloroplasts and their membranes are the most sensitive plant organelle to water stress [50, 51] and low temperatures [18, 52, 53]. Given the nature of hairy roots, in which tissue cells with chlorophyll are not present, and therefore are not photosynthetic organs, could be the reason why *E*. *acanthocarpum* hairy roots seem not to be much affected by water or osmotic stress conditions, and the increment of TL at the initial sampling times might be due to a larger cell division rate in these meristematic tissues, accompanied by a major formation of cytoplasmic membranes and production of PUFA [54].

In order to study the effect of both decreasing the culture temperature from 25 to 15°C and the presence of other stress agents in *E. acanthocarpum* hairy roots, in lipid composition, sampling points 4 and 5 were taken as the most representative ones by making a compromise between biomass production and the rate of FA production [40]. Lipid class composition was affected by both, the temperature and the presence of sorbitol and glucose in the nutrient medium, although generally no significant differences were observed between the values of lipid classes PC, PS + PI, and PE and total polar and neutral lipids (Tables 2(a) and 2(b)). Among polar lipids, phosphatidylcholine (PC), phosphatidylserine, and phosphatidylinolsitol (PS + PI) ranged from 8.53 to 9.13% and 4.51 to 6.17% of the total lipids for PC and PS + PI, respectively, in cultures B1 and C1 (Table 2(a)). Different authors propose that under water stress conditions, free FA are produced most abundantly by the action of lipases over polar lipids and subsequently stored in esterified triacylglycerols (TAG), increasing thereby the neutral lipids. Furthermore, the polar lipids rise may be explained because water stress increases the synthesis of new membranes [55]. In the same fashion, it has been observed in roots grown at low temperatures how total content of polar lipids was augmented twice its initial value at room temperature [56, 57]. 

There was a clear increasing tendency of phosphatidylglycerol (PG) in *E. acanthocarpum* cultures grown at 15°C, going from an average of 3.48% of TL at 25°C (culture B1, Table 2(a)) to 8,25% of the TL at 15°C (culture C1, Table 2(a)). The rise of PG in *E. acanthocarpum* hairy roots in response to a reduction of temperature is consistent with several studies where the content of PG in roots of *Avicennia germinans* was higher than in those plants more resistant to low temperatures [58]. In another study carried out on the plasma membrane of wheat seedlings, a rise of all classes of phospholipids, including PG, was recorded when the culture temperature was 2°C [59]. It has also been speculated that the direct relationship between temperature and richness of PG in plant tissues is due to the glycerol-3P-acyltransferase specificity, responsible for the esterification of certain FA, usually 18:0, 18:1, and 16:0 at sn-1 position of PG and also due to its response to temperature [60, 61]. No significant changes were observed in PE in B1 and C1 cultures, with percentages ranging from 10.75 to 12.18% of TL. Finally, when calculating the total values of polar and neutral lipids, no drastic changes were observed, with values of 29.70 to 33.39% and from 59.59 to 64.05%, respectively (Table 2(a)). After studying the values for each lipid class in the other cultures (C2–C4), and compared to those profiles obtained in C1, no significant changes were detected, except for the case of PG, reaching 13.77% of TL in culture C2 (Tables 2(a) and 2(b)). There are very few studies addressing the distribution of lipid classes in roots, although one describes the PG content in the thylakoid membranes and its relative increase, together with unsaturation enrichment of FA esterified in PG against low temperatures, both in monocotyledons and dicotyledons [61, 62]. Also, it was reported that there is an enrichment of the saturated 16:0, 18:0 FA, constituting about 40% of those esterified FA in PG, and the monoene trans-16:1, in plant species sensitive to low temperatures, while the wealth of saturated FA in resistant species to low temperatures decreased to 20% [63]. These data suggest a direct relationship of this phospholipid with plant resistance to low temperatures, although other factors providing such feature might also exist [64].

The distribution of lipid classes in *E. acanthocarpum* hairy roots was similar to that of a nonphotosynthetic tissue, exhibiting high polar lipid values, mainly PE and PG, not recording the typical photosynthetic tissue lipids, such as monogalactosyl diacylglycerides (MGDG) and digalactosye diacylglycerides (DGDG), present in chloroplasts.

**Figure 3:**
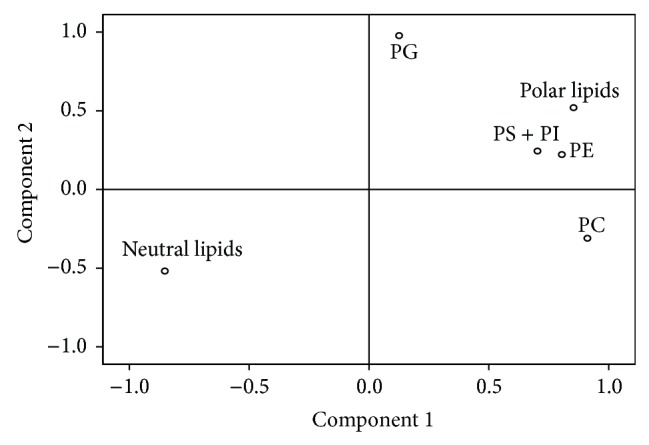
Factor loading plots of the principal component analyses of the percentages of lipid classes in *Echium acanthocarpum* E1.5 cell line hairy roots, growing in different media at 15°C (C1–C4 cultures). PC = phosphatidylcholine; PS + PI = phosphatidylserine and phosphatidylinositol; PG = phosphatidylglycerol; and PE = phosphatidylethanolamine.

In order to carry out the statistical analysis of the data, variables for cultures B1 and C1, sharing the same culture medium, were analysed to observe how temperature (25 and 15°C) affects the distribution of lipid classes. Only significant differences for PG were observed, whose percentage was the highest when grown at 15°C (Tables 2(a) and 2(b)). Secondly, in order to study the influence of the use of glucose and 0.2 M sorbitol in the nutrient medium and the time of sampling, a principal component analysis (PCA) to the percentages of the obtained lipid classes for C1–C4 cultures was performed (Table 3). We obtained two principal components (PC1 and PC2), which explained 85.68% of the variance. PC2 (19.78% of variance) was positively correlated with the PG, while PC1 (65.91% of variance) was positively related to PC, PS + PI, PE, and total polar lipids, while it was negatively correlated with the neutral lipids (Table 3, Figure 3). 

Accordingly, it could be assumed that PC1 is correlated with the richness of polar lipids and PC2 reflects the amount of PG in hairy root tissues.

After removal of the principal components PC1 and PC2, we conducted a two-way ANOVA test in order to determine whether the studied factors, that is, stressing factors and sampling points and the interaction between them, had influence on the extracted new variables PC1 and PC2 (Table 4).

**Figure 4:**
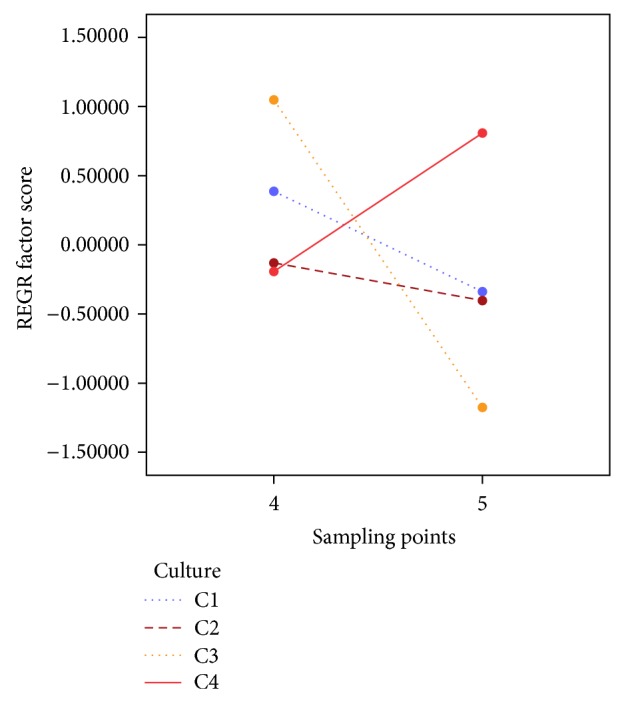
REGR factor score for PC1 depending on sampling point 4 or 5 and categorized by type of culture (C1–C4), showing the interaction effect of abiotic stress and time.

Only when both factors interacted for PC1, the level of significance was less than 0.05 (Table 4, Figure 4); that is, polar lipids usually remained constant. Conversely, the PC2 variable, reflecting the content of PG in the sample, (Figure 3, Table 3) was influenced by the presence of sorbitol or glucose in the nutrient medium (Table 4). Interestingly, it was observed that PG content was significantly higher in C2–C4 cultures, those in which 0.2 M sorbitol or 3% glucose was present. These data are consistent to those observed in *A*. *thaliana* leaves reporting a nonsignificant increase of this class of lipids; moreover, when water stress conditions became more severe, a PG decrease and a pronounced increase of typical lipid classes of green tissues (MGDG and DGDG) were observed as well as ALA (18:3n-3) in PC lipid class and DGDG [48]. Likewise, it was reported the same PG increase in *Pachyrhizus ahipa* leaves [49]; furthermore, in barley roots grown in the presence of 100 mM NaCl, PG reached around 5% of TL [65] and, in *Carthamus tinctorius* leaves, where PG and the set of polar lipids especially PC rose under smooth water stress conditions but decreased significantly when water conditions became more acute [55]. On the other hand, PG content was not influenced by time or sampling point or by the interaction of both in the culture (Table 4).

**Figure 5:**
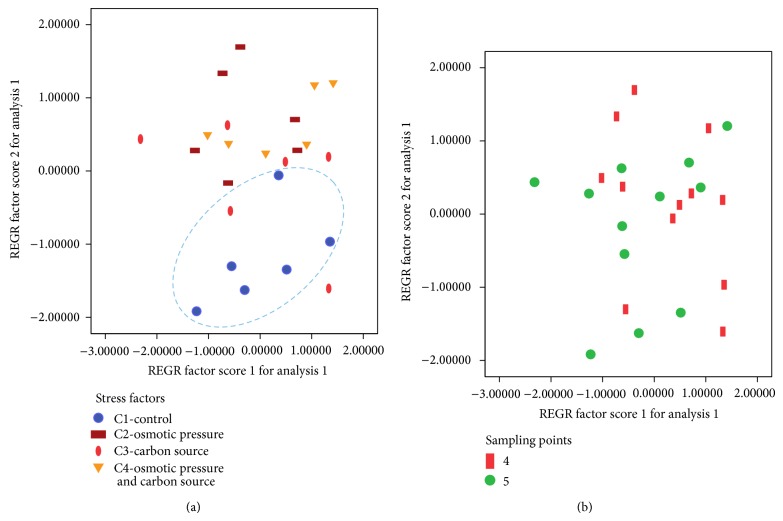
(a), (b) Plot of *Echium acanthocarpum* hairy root samples in terms of the principal components PC1 and PC2 stratified according to (a) cultures (C1–C4) or (b) sampling points.

Therefore, when samples were plotted as a function of the main components and stratified according to the presence of stressing factors, a clear separation between the control group, culture C1, or the other cultures (C2–C4), also grown at 15°C, was observed (Figure 5(a)). When these samples were stratified according to the sampling points, an overlap between the groups was observed (Figure 5(b)).

### 3.3. Effect of Stress on the Unsaturation Degree of the Fatty Acids

The FA detected in *E. acanthocarpum* hairy roots were the same as those reported previously [36], but the percentages of each FA changed considerably. Thus, the following saturated FA were detected: palmitic acid (16:0) and stearic acid (18:0), along with other minor FA like 20:0, 22:0, and 24:0 (Tables 5 and 6). Similarly, high percentages of the monounsaturated FA 18:1n-9 and 18:1n-7 were measured, with a total of approximately 28% of saturated FA, with 6% of monoenes in all samples. The more characteristic polyunsaturated n-6 FA, LA and GLA, represented 50–55% of the total FA. Furthermore, the n-3 PUFA, ALA and SDA, represented 6–9% of all FA, depending on the cultures (Tables 5 and 6). 

Again, sampling points 4 and 5 were the most representative, according to the biomass reached and the FA profiles displayed [36, 38]. 

#### 3.3.1. Fatty Acid Profile of Culture C1 of *Echium acanthocarpum* Hairy Roots

Analyses and comparison of the data of FA profiles and the different indeces were performed after one-way ANOVA test. The FA profile appeared very homogeneous even after 55 days of culture (Table 6, Supplementary Tables S1–S4 in Supplementary Material available online at http://dx.doi.org/10.5402/2013/169510 additional information). There were only a few statistical differences along the different sampling points, mainly regarding the percentages of minor saturated FA as well as SDA. The 14:0 was significantly more abundant at sample point 1 (0.26% of total FA), while 18:0 was more abundant at sampling points 1 and 2 (2.61% and 1.83 of total FA). However, values for 24:0 were significantly higher at the end of the culture recording 2.70% of total FA (Tables S1–S4 additional information).

**Figure 6:**
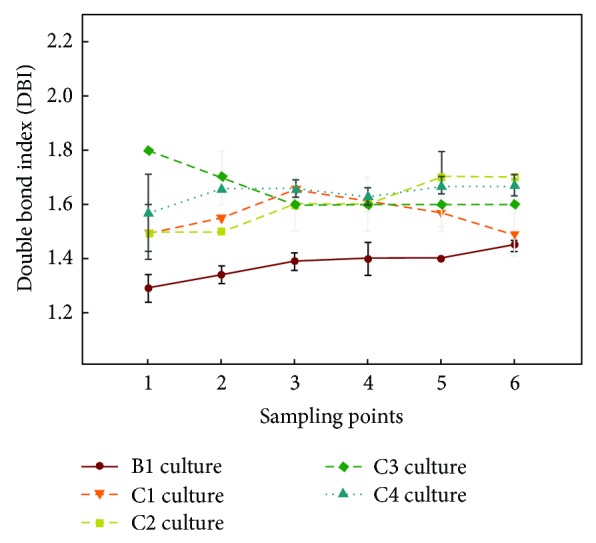
Double bond index (DBI) in *Echium acanthocarpum *hairy roots cultured in different growth media at two temperatures. DBI was calculated as [(% 18:1n) + 2 × (% 18:2n) + 3 × (% 18:3n) + 4 × (18:4n)]/100.

 Regarding the main PUFA present in the samples, that is, LA (18:2n-6) and GLA (18:3n-6), no significant differences were observed (Tables 5 and 6; S1–S4 additional information). LA values were slightly higher at sampling point 1, with a total of 38.11% of the total FA, although this difference was not significant. Regarding the other sampling points, LA values ranged from 34 to 36%. The n-6 Δ6-desaturated FA, GLA, reached slightly higher values in the middle of the culture (17.51 to 19.23%), corresponding to the exponential phase of the growth curve, although these differences were not statistically significant (Tables 5 and 6; S1–S4 additional information). With regard to the n-3 PUFA, ALA showed 5-6% of total FA, almost constant over time (Tables 5 and 6; S1–S4 additional information). On the other hand, SDA showed 1.62 to 2.12% of total FA, being its percentages significantly higher along sampling points 3–6 (Tables 5 and 6; S1–S4 additional information). It is well known that biological membranes adjust their composition according to the environmental conditions [66, 67], and they are also the most susceptible part of the cell to temperature decline, requiring to adjust their fluidity and permeability to maintain membrane functionality [68], which is achieved basically by increasing FA unsaturation degree [11, 13, 45, 69–72] and in particular augmenting those FA with three unsaturations, reporting a rise in 18:3n-3 (ALA), at the expense of declining 18:2n-6 (LA), depending on plant species characteristics [21, 45, 61, 71, 73–78]. In the case of *E*. *acanthocarpum* hairy roots, the rise of unsaturations was mainly due to the increase of Δ6-desaturated FA, in particular GLA, also supported by the general richness in double bonds of the FA samples as shown by the double bond index (DBI) data, which was clearly augmented when reducing the culture temperature from 25 to 15°C (Figure 6), similar to other reports [45, 49, 72, 75, 79].

Although very few statistical differences were recorded for the FA contents, surprisingly, the analysis of the different calculated indexes and ratios showed significant differences for most of them along the different sampling points. The (n-6) Δ6-desaturation index showed slightly higher values of approximately 0.34, at sampling points 4 and 5 (Tables 5 and 6), parallel to higher values observed for GLA, which were approximately 17-18% of total FA. Likewise, significant differences were also found for the other Δ6-desaturation index, (n-3), which was maximum, 0.27 to 0.29, also at sampling points 4 and 5 and paralleled with changes in SDA (Tables 5 and 6). In addition, the evolution of the DBI was studied, whose values were significantly higher among sampling points 2–5 (1.55 to 1.65) (Figure 6, Tables 5 and 6; S1–S4 additional information).

**Figure 7:**
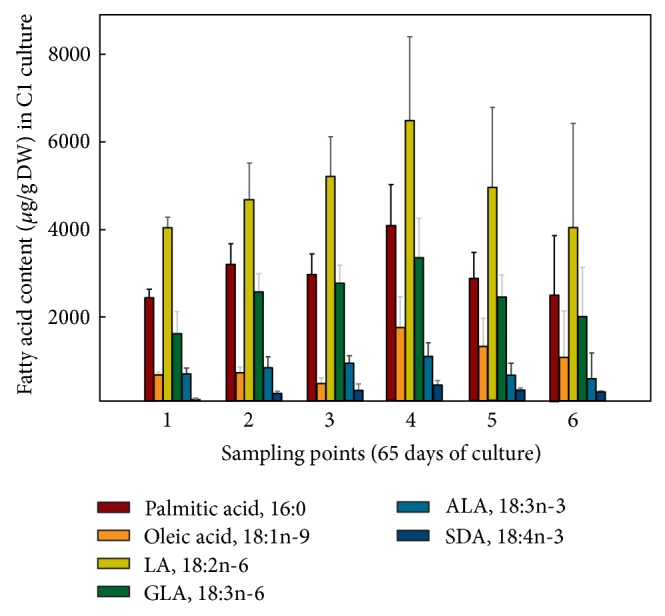
Absolute amounts of different fatty acids (*μ*g/gDW) present in* Echium acanthocarpum *hairy roots grown in B5 medium, 3% sucrose, 1% PVP at 15°C (culture C1). Each value is the mean ± standard deviation of three replicates.

**Figure 8:**
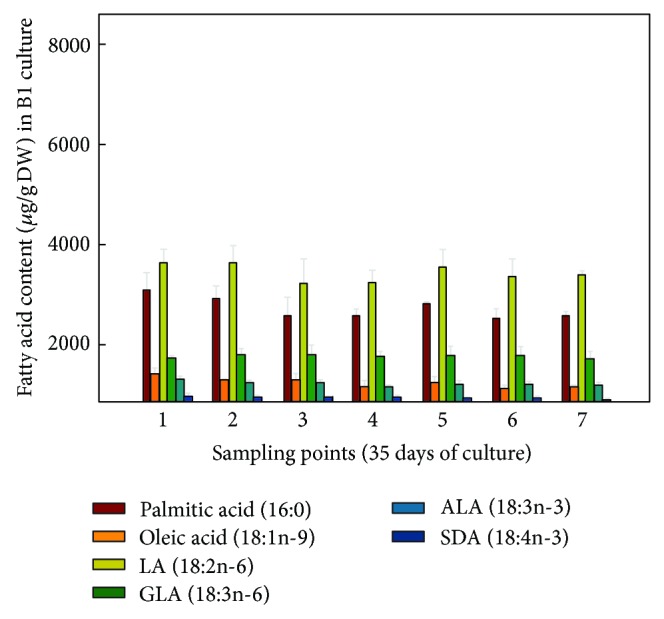
Absolute amounts of different fatty acids (*μ*g/gDW) present in* Echium acanthocarpum *hairy roots grown in B5 medium, 3% sucrose, 1% PVP at 25°C (control culture B1). Each value is the mean ± standard deviation of three replicates.

When the FA contents were expressed in absolute values, it was observed for the hairy roots grown at 15°C (Figure 7) that LA was the most abundant FA, 6,473.82 *μ*g/gDW at sampling point 4, representing a 2.15-fold increase compared to culture B1 grown at 25°C, with a minimum value of 4,007.68 *μ*g/gDW at sampling point 1. GLA was also very abundant, 3,316.92 *μ*g/gDW also at sampling point 4, a 3.31-fold increase compared to culture B1 grown at 25°C, with a minimum of 1,594.88 *μ*g/gDW at sampling point 1. More importantly, the FA of the n-3 series, ALA reached 1,046.32 *μ*g/gDW, representing a 2.75-fold increase compared to culture B1 grown at 25°C and SDA 391.07 *μ*g/gDW, a remarkable 3-fold increase compared to culture B1 grown at 25°C, both at sampling point 4 (Figures 7 and 8). Due to their importance and abundance in the sample, values of 16:0 and 18:1n-9 FA were also interesting. The maximum amounts of 16:0 (4,031 *μ*g/gDW) and 18:1n-9 (1,715 *μ*g/gDW) were recorded at sampling point 4, with lower values at other sampling points (Figure 7). 

As quoted above, these values were much higher than those achieved by the same hairy roots but grown at 25°C (culture B1, control) (Figure 8), whose maximum values were 3,000 and 1,000 *μ*g/gDW for LA and GLA, respectively; moreover, FA of the n-3 series were recorded in lower quantities; ALA 380 *μ*g/gDW (sampling point 5) and SDA 130 *μ*g/gDW (sampling point 1) (Figure 6). 

Another possible explanation to the increase in PUFA would be given precisely by the direct influence of temperature decrease on the expression of different desaturase coding genes, transcriptional, translational or posttranscriptionaly. This fact was observed for the first time in soybean [22] and later in cyanobacterium [80], followed by others reports [7, 23, 66, 81–83]. Moreover, in rice, a different regulation at the transcriptional level of two desaturases (FAD7, FAD8) depending on culture temperature was reported [84, 85]. This regulation is so effective that *Arabidopsis* mutants that lacked the FAD5 desaturase and being deficient in 16:1 desaturated products showed the same FA profiles compared with wild plants at the same temperature, with the levels of 18:2n-6 remaining constant, demonstrating the importance of this FA in plasma membrane homeostasis [70]. In *E. acanthocarpum* hairy roots, values of Δ6-desaturation index were higher when culture temperature decreased, suggesting that gene expression and/or the Δ6-desaturase activity itself may be temperature dependent although other influences may also take place.

#### 3.3.2. Fatty Acid Profile of Culture C2 of *Echium acanthocarpum* Hairy Roots

Analogously, the same parameters and ratios were evaluated when hairy roots were grown in culture C2, with the addition of 0.2 M sorbitol, as osmotic agent, and also at a temperature of 15°C. FA profiles vaguely differed from those observed for culture C1, although significant differences along the sampling points were found for minor FA, such as stearic acid (18:0), which showed maximum values at sampling points 1 and 2 (Tables S1-S2 additional information), with percentages of 2.62 to 3.11 of total FA. There were also statistically significant differences for 18:1n-7, exhibiting also higher values in the first three sampling points, with percentages of 1.01 to 1.33. (Tables S1–S3 additional information). Furthermore, the more unsaturated FA studied, that is, GLA and SDA, showed lower percentages at sampling point 1, 12.72 and 0.77, respectively, with maximum values of 3% for SDA and 19% for GLA at sampling points 3–6 (Tables 5 and 6). In regard to LA and ALA, their values were relatively constant throughout the culture, with values of 33.98 to 39.10% for LA and 5.85 to 7.33% for ALA.

The n-6 Δ6-desaturation index showed significant differences, with a slightly lower value (0.25) at sampling point 1, corresponding to the lowest value of GLA (12.72%) (Table S1 additional information). Contrarily, the highest values were observed at sampling points 4-5 (Tables 5 and 6), ranging from 0.36 to 0.37, respectively, although they were not statistically different from the other values. By contrast, the n-3 Δ6-desaturation index showed significant differences, with maximum values of 0.29 to 0.32, at sampling points 4–6 (Tables 5 and 6; S3 additional information); again, these data paralleled the SDA variations. Finally, the DBI showed no significant differences, with values being higher at later culture time, coinciding with the stationary phase (1.63 to 1.69) (Figure 6).

**Figure 9:**
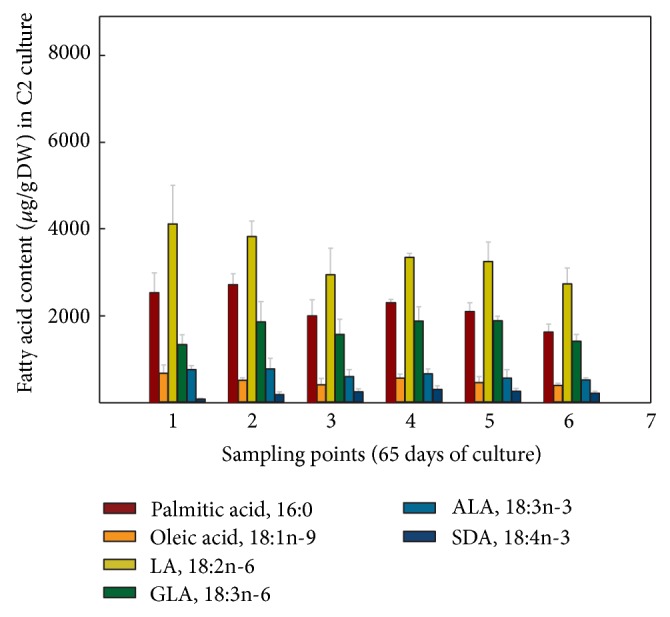
Absolute amounts of different fatty acids (*μ*g/gDW) present in *Echium acanthocarpum *hairy roots grown in B5 medium, 3% sucrose, 0.2 M sorbitol, 1% PVP, at 15°C (culture C2). Each value is the mean ± standard deviation of three replicates expressed.

In terms of absolute values, the highest amounts of both Δ6-desaturated FA, GLA and SDA, 1,880 and 303 *μ*g/gDW, respectively, were obtained at sampling point 4 (Figure 9), being slightly lower than those obtained in nonsorbitol containing culture C1 (Figure 7). Again, LA was the most abundant FA, displaying 4,114 *μ*g/gDW at sampling point 1. Besides, the maximum value of ALA was 771 *μ*g/gDW at sampling point 2, which was also lower than that recorded in culture C1 (Figures 7 and 9).

Therefore, comparing the results obtained with culture C1 (at 15°C) with those of culture C2 with the addition of sorbitol (osmotic stress) also grown at 15°C, the combination of these two factors did not further increase the yield of the important Δ6-desaturated FA under investigation, that is, ALA, SDA, LA, and GLA; moreover, the lower amounts of FA recorded in culture C2 well correlate to the lower percentage of total FA in the lipid extract (Tables 5 and 6).

#### 3.3.3. Fatty Acid Profile of Culture C3 of *Echium acanthocarpum* Hairy Roots

When hairy roots were grown in the presence of 3% glucose instead of 3% sucrose also at 15°C, similar FA values were observed; although, in general, significant differences were less frequent. Contrarily to the yield in culture C2, here SDA was significantly more abundant at sampling point 1, reaching an attractive 3.45% of total FA (Table S1, additional information), which was statistically similar to those recorded at sampling points 3–6, ranging from 2.37 to 2.77% of total FA (Tables 5 and 6; S2, S4 additional information). For the rest of PUFA present in the samples, the percentage values were statistically similar throughout time. LA exhibited total amounts of 31.95 to 35.93% of total FA, while ALA reached amounts from 5.87 to 8.30%, with GLA showing similar values to those achieved by the other cultures, from 17.58 to 20.13% of total FA (Tables 5 and 6).

In this culture, the ratio n-3/n-6 showed a significantly higher value (0.21) at sampling point 1, due to the increased in SDA at the same data point, which was 3.45% (Table S1 additional information). For the remaining data points, the value of this index was 0.17, except at sampling point 6 (0.15) (Tables 5 and 6; S2–S4 additional information). It was also observed that the value for n-3 Δ6-desaturation index was significantly lower (0.22) at sampling point 2 but ranging from 0.29 to 0.32 at other sampling points (Tables 5 and 6; S2–S4 additional information). Likewise, significant differences on DBI were observed with a minimum value of 1.55 again at sampling point 2 but between 1.59 and 1.75 for the other time points (Figure 6).

**Figure 10:**
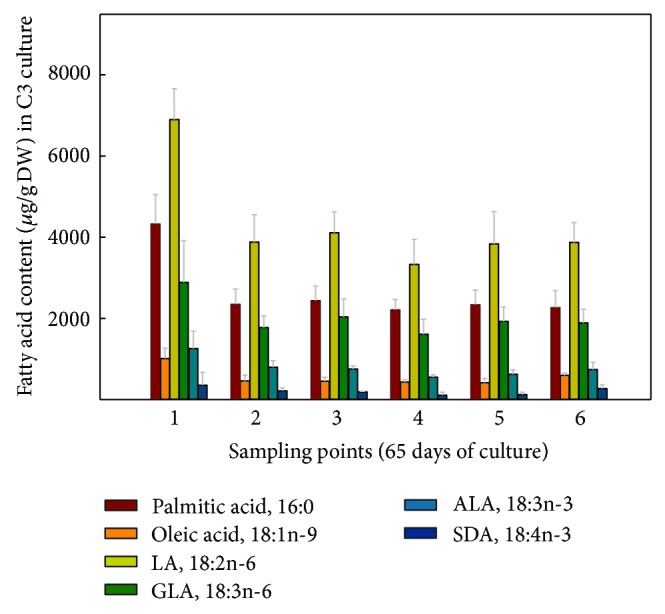
Absolute amounts of different fatty acids (*μ*g/gDW) present in *Echium acanthocarpum *hairy roots grown in B5 medium, 3% glucose, 1% PVP at 15°C (culture C3). Each value is the mean ± standard deviation of three replicates.

Interestingly, FA values expressed in *μ*g/gDW showed maximum PUFA amounts at sampling point 1 (Figure 10), due in part to the greater amount of TL extracted at this point (Figure 2). Furthermore, at sampling points 3–5, PUFA amounts remained very stable, LA, 4,449–4,997; ALA 809–885; GLA, 2,388–2,724; and SDA of 369–389 *μ*g/gDW; that is, values are slightly lower than those obtained for cultures C1-C2.

#### 3.3.4. Fatty Acid Profile of Culture C4 of *Echium acanthocarpum* Hairy Roots

Finally, hairy roots were grown in a medium containing 0.2 M sorbitol and 3% glucose and also at 15°C. In general, the same trends in terms of percentages recorded for each FA were observed. Significant differences were detected for some saturated FA with palmitic acid (16:0) and stearic acid (18:0) showing lower values at the last sampling point, with 21.66% and 1.81% of total FA, respectively (Tables 5 and 6; S4 additional information). The major PUFA showed no significant differences, with LA being slightly more abundant than for the other cultures (C1–C3) grown at 15°C, with values ranging from 36.70% to 38.08% of total FA (Tables 5 and 6; S1–S4 additional information). ALA, however, appeared to maintain the same percentages as for the other cultures, representing 6.16% to 7.75% of total FA. The most unsaturated PUFA in the samples, GLA showed interesting percentages at sampling points 4 and 5, with values between 18 and 19% of total FA (Tables 5 and 6), while SDA, similar to C3 culture, which differs from C4 on the absence of osmotic pressure, showed higher values at the beginning of the culture, up to 2% of total FA and at the end of culture, where it reached 2.53% of total FA (Tables 5 and 6; S1–S4 additional information).

The different ratios and indexes studied did not show any statistical differences, with a maximum value of 0.34 (Table 6) for n-6 Δ6-desaturation index at sampling point 5 and 0.21 and 0.26 at sampling points 2 and 6, respectively, for the n-3 Δ6-desaturation index (Tables 6; S1–S4 additional information). The n-3/n-6 ratio showed values of 0.13 to 0.18 throughout the culture, and DBI index showed 1.56 to 1.67 (Figure 6).

**Figure 11:**
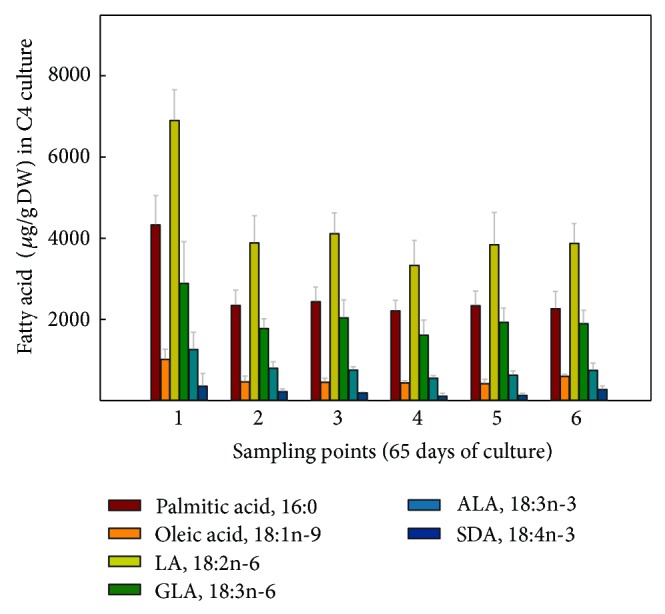
Absolute amounts of different fatty acids (*μ*g/gDW) present in *Echium acanthocarpum* hairy roots grown in B5 medium, 3% glucose, 0.2 M sorbitol, 1% PVP at 15°C (culture C4). Each value is the mean ± standard deviation of three replicates.

The absolute amounts of each FA displayed maximum values of PUFA at the first sampling point (Figure 11), corresponding also to the highest TL content (Figure 2). Meanwhile, for the remaining sampling points, TL was lower, and therefore the amounts of each FA also decreased, showing the following values: LA 3,335–4,112; ALA 555–795; GLA 1,614–2,036; and for SDA 112–215 *μ*g/g DW (Figure 11). These values were slightly lower than those recorded for cultures C1 and C3 but higher than those exhibited in culture C2. Moreover, here in culture C4, lower TL values were recorded, being significantly different compared to the other cultures at sampling point 4 (Figure 2), which would justify the lower FA amounts present in this culture parallel to the lower TL content, especially when compared with culture C1.

**Figure 12:**
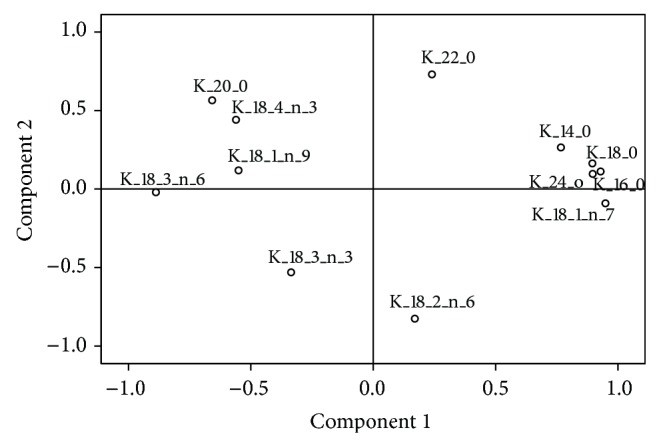
Factor loadings for the percentages of fatty acids in *Echium acanthocarpum *hairy root cultures B1 and C1 grown at 25°C and 15°C, obtained after principal component analysis.

The influence of culture temperature (25 versus 15°C) on the unsaturation degree in the pool of FA was studied by principal component analyses, which allow to reduction of the dimensionality of the variables. The statistical variables (12 different FA) were introduced for the cultures B1 and C1, since the only difference between these cultures was the temperature at which hairy roots had been grown, 25°C and 15°C, respectively, with culture B1 selected as control. Two principal components (PC1 and PC2) were extracted, which explained 67.88% of the variance and were able to summarize the information contained in the 12 variables (FA). PC1, 48.95% of the variance, was positively correlated with the minor saturated FA 14:0, 18:0, and 24:0, with 16:0 and the monoene 18:1n-7 (Table 7, Figure 12). In addition, PC1 was also found to be correlated, although negatively, with the determinant FA involved in the PUFA metabolic pathway, that is, oleic acid (18:1n-9) and the Δ6-desaturated acids, GLA and SDA (Figure 12; Table 7). Moreover, PC2, 17.92% of variance, was negatively correlated with the precursors of these FA, that is, LA and ALA, and positively correlated with 22:0 (Figure 12; Table 7). 

Accordingly, it could be said that PC1 is again correlated with the abundance of saturated FA and inversely proportional to the adaptation of hairy roots to temperature, meaning that higher PC1 values suggest a decrease in GLA and SDA and a lower relative activity of the Δ6-desaturase enzyme. On the other hand, and also inversely, PC2 reflects PUFA contents which are the substrates of the Δ6-desaturase, that is, LA and ALA. The same correlations were established when performing a principal component analysis to the absolute values of FA.

In order to test whether the studied factors, temperature and time of culture, and the interaction between them, would affect the obtained variables, PC1 and PC2, a two-way ANOVA test was performed. As shown in Table 8, the level of significance indicated that both temperature and time had influenced PC1, while PC2 was only influenced by the interaction of both factors. Furthermore, when plotted, the samples were clearly separated according to the main components and stratified with the temperature (Figure S1(a) additional information). In contrast, when the same stratified representation was made regarding sampling time, no effect was observed and the groups clearly overlapped (Figure S1(b) additional information). However, the influence of time on PC1 when plotted against sampling points was strongly attenuated between sampling points 3 and 5 (Figure S2 additional information).

Following the establishment of a direct relationship between the decrease of culture temperature and the enrichment of PUFA in the sample, the possible influence of the carbon source and sorbitol on the FA was also analyzed. A new principal component analysis was performed, and different variables (FA) for cultures C1, C2, C3, and C4, grown at 15°C in the presence of osmotic stress (cultures C2 and C4) and 3% glucose as carbon source (C3 and C4), were analysed, choosing culture C1 as a control which also grew at 15°C but without sorbitol or glucose. 

**Figure 13:**
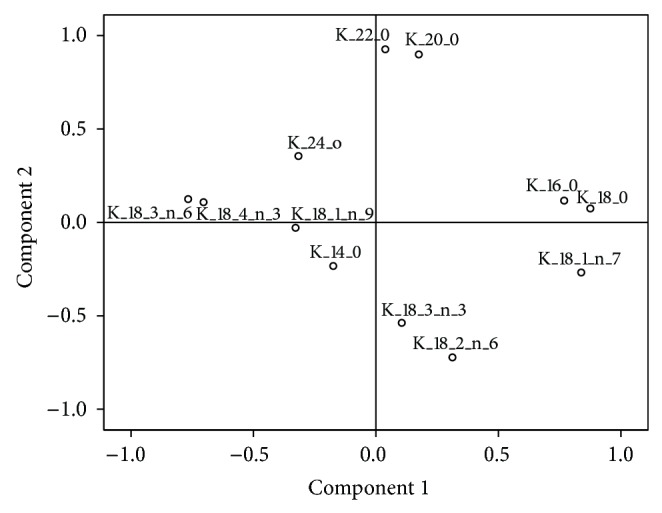
Factor loadings obtained after principal component analysis, for the percentages of fatty acids in *Echium acanthocarpum *hairy root cultures C1–C4 grown at 15°C.

The principal components analysis of the percentages of FA identified two new components PC1 and PC2, which explain 52.50% of the data (Figure 13, Table 9).

PC1 (30.77% of variance) was positively correlated with the saturated FA, 16:0 and 18:0, and with the monoene 18:1n-7. Furthermore, PC1 was negatively correlated with the more unsaturated FA and products of the Δ6-desaturase enzyme activity, GLA and SDA. Whereas PC2 (21.73% of the variance) was strongly and negatively correlated with LA and ALA, both Δ6-desaturase enzyme substrates, and was also associated with some minor saturated FA, 14:0, 20:0, 22:0, and 24:0. In other words, high values of the new PC2 variable suggest lower amounts of the Δ6-desaturase enzyme substrates (LA and GLA) and, possibly, an increase of the FA product of this enzyme (GLA and SDA). The PCA performed with the absolute amounts of FA also showed the same correlations.

A two-way ANOVA test showed a significant influence of both the stressing factors present in the cultures and factor time on PC1 and PC2 as well as the interaction between the two on PC1 (Table 10). When plotting the data of the samples based on PC1 and PC2 and stratifying them according to the stressing factors (different cultures), a separation between the groups was observed, especially for those samples belonging to culture C4 (Figure S3 additional information), in which osmotic stress was applied by the presence of sorbitol. In contrast, when plotting the data stratified according to sampling points, a greater overlap between the groups was also observed (data not shown).

Post-hoc analysis performed for each of the principal components revealed that cultures pairs C1-C2 and C3-C4 were statistically equal for PC1 (Figures S4(a)–4(d) additional information). The lowest PC1 values corresponded with culture C1 (control) and culture C3, in which 3% glucose was added as carbon source, indicating that these cultures showed, in general, less content of saturated FA, a higher content of the PUFA, GLA and SDA, and a relatively higher Δ6-desaturase activity.

Moreover, for PC1, the values were significantly higher at sampling points 1 and 2 (Figure S4(b) additional information), and points 3–5 appeared more favorable in order to obtain a more abundant set of PUFA. The PC2 post-hoc study displayed significant differences in culture C4 (Figures S3, S4(c) additional information). Considering that PC2 was a reflex of the low content of the Δ6-desaturase substrates, LA and ALA, culture C4 would be the one containing the largest amounts of these two FA. This theoretically suggests a lower Δ6-desaturase activity and, therefore, fewer amounts of the PUFA, GLA and SDA, being in full agreement with the results observed for PC1. Regarding the influence of the sampling points on PC2, a parity between the sampling points 1–4 and 2–6 was observed, with sampling points 4-5 appearing as the most appropriate since these showed a high percentage of the target PUFA of this study, GLA and SDA (Figure S4(d) additional information), and the tendency which suggests that sampling points 4 and 5 would be the most appropriate because of the high TL content (Figure 2).

Under water stress conditions, which is mimicked in *in vitro* cultures by applying osmotic stress, reports do not show an overall increase of unsaturation, even more a decrease of 18:3n-3 was found, the most unsaturated FA present in the plant species studied which was a reflex of the damage caused by stress [48, 49, 86]. When water stress conditions become more severe, this FA percentage can be increased, as in the experiments conducted in the leaves of *A. thaliana *[48].

In contrast, in other cases, the use of osmotic stress or high osmotic pressure to stimulate the production of secondary metabolites in plant cell cultures appeared as an effective strategy [25–31]. A possible explanation of this fact could be that stress conditions inhibit initially cell growth and division and, hence, increase the concentration of cell culture carbohydrates, thus being a more available resources for secondary metabolism [30]. However, in *E. acanthocarpum* hairy roots, no significant increase or decrease in fresh weight of cultures C2 and C4 was registered, which may correlates with this hypothesis (Figure 1).

In ginseng roots, the added sorbitol may act by activating the phenylalanine amino-lyase enzyme, increasing the production of reactive oxygen species (ROS). ROS are also closely related to the response of plants to low temperatures [11]; however, the presence of ROS was not studied in our research.

In *E. acanthocarpum* hairy root system, the combined possible effects produced at low temperature and osmotic stress do not seem to be cumulative on the FA profile. Regarding the influence of the carbon source on the FA profile, it is necessary to note that, in plants FA synthesis occur predominantly in plastids and require a carbon source, ATP, and reducing power. The carbon source for the FA synthesis is usually in the form of acetyl-coenzyme A (acetyl-CoA), which is not able to cross the plastid membrane. Thus, precursors of acetyl-CoA should be generated inside the plastids or be imported from the cytosol. In heterotrophic seeds, lipids may accumulate in large quantities, for example, comprising up to 40% dry weight of the embryo, as in sunflower seeds, forcing a massive influx of carbon source into the plastids to maintain synthesis [87]. Thus, various studies have shown that a wide range of cytosolic metabolites, such as glucose-6-P, phosphoenolpyruvate (PEP) and pyruvate malate, are capable of withstanding the FA biosynthesis [9]. Moreover, the ATP required for the synthesis of FA in nonphotosynthetic tissues, such as *E. acanthocarpum* hairy roots, occurs in the plastids, from the synthesis of acetyl-CoA, glucose-6P, and PEP [88, 89]. Despite this fact, *in vitro* studies of plastids, isolated from sunflower seeds, reported that malate was the carbon source supporting a better FA biosynthesis, although these results were not in agreement with those obtained *in vivo*, where malate was the minor source [9, 87]. A more recent study, which used corn embryos, showed that the main carbon source for the synthesis of FA was PEP originated from hexose-P [90, 91]. These authors also suggested that the glucose dehydrogenase-6-phosphate played an important role in the provision of the reducing power in the form of NADPH for FA biosynthesis in seeds, where photosynthesis is restricted.

## 4. Conclusions

The results presented here show the usefulness and high potential of the *E. acanthocarpum *hairy roots for biosynthesizing and accumulating a large range of polyunsaturated FA, including the target SDA and GLA in appreciable quantities; furthermore, from the point of view of production and accumulation of SDA and GLA, *E. acanthocarpum *hairy roots are more efficient and boosted PUFA yields when grown in a nutrient medium consisting of B5 basal salts, sucrose or glucose as a carbon source and more importantly at a temperature of at least 15°C and for a period of not less than 45–55 days (sampling points 4 and 5), resulting in a drop of LA but a strong rise of SDA (60%) and more moderate for GLA (35%). On the other hand, the application of osmotic stress did not seem to exert a positive influence on the amount of PUFA accumulated, and the combination of a lower culture temperature and glucose did not show a cumulative boosting effect on PUFA production; although this carbon source was similarly attractive proving the appropriateness of the strategy of applying abiotic stress in this novel culture system to enhance FA yields. Using this culture system, further research results are being evaluated on the effects of the overexpression of the Δ6-desaturase gene from *Primula vialii*, which specifically catalyzes the conversion of ALA into SDA, after applying also the abiotic stress conditions which yielded the highest results presented here.

## Supplementary Material

The additional information accessible on-line provides data presented in tables and figures of the different parameters recorded in this investigation at the other sampling times of the different culture growth curves, as well as additional data of the statistical analyses conducted and not present in the printed version of the publication.

## Figures and Tables

**Table 1:** Liquid culture media employed for the growth and fatty acid studies of *Echium acanthocarpum* hairy roots grown under different stress conditions. B5 [8].

Name	Basal salt	Carbon source	PVP	Sorbitol	Temperature
Culture B1	B5	3% sucrose	1%	—	25°C
Culture C1	B5	3% sucrose	1%	—	15°C
Culture C2	B5	3% sucrose	1%	0.2 M	15°C
Culture C3	B5	3% glucose	1%	—	15°C
Culture C4	B5	3% glucose	1%	0.2 M	15°C

PVP: polyvinyl pyrrolidone.

**Table:** (a) Data of sampling point 4

Sampling point 4	Culture B1	Culture C1	Culture C2	Culture C3	Culture C4
TL (mg/g DW)	33.41 ± 3.66^ab^	41.67 ± 2.45^b^	35.05 ± 4.50^ab^	32.20 ± 4.94^ab^	31.03 ± 1.03^a^
PC	9.30 ± 1.37	8.53 ± 1.34	6.46 ± 1.81	10.13 ± 1.51	6.50 ± 1.18
PS + PI	6.17 ± 0.67	4.51 ± 4.09	5.82 ± 2.11	6.92 ± 2.09	3.05 ± 1.68
PG	3.48 ± 1.42	8.25 ± 1.70^*^	13.77 ± 2.36	9.12 ± 2.32	12.54 ± 1.56
PE	10.75 ± 0.62	12.18 ± 1.49	9.80 ± 1.16	11.02 ± 0.77	12.72 ± 4.51
Polar lipid	29.70 ± 2.77	33.49 ± 5.93	35.85 ± 3.54	37.19 ± 2.89	34.81 ± 8.21
Neutral lipid	64.05 ± 5.67	59.59 ± 5.34	59.97 ± 2.44	59.04 ± 6.20	65.19 ± 8.21
unknown	6.25 ± 2.92	6.92 ± 2.21	4.17 ± 1.11	3.77 ± 3.68	13.43 ± 4.64

**Table:** (b) Data of sampling point 5

Sampling point 5	Culture B1	Culture C1	Culture C2	Culture C3	Culture C4
TL (mg/g DW)		35.58 ± 7.33	28.59 ± 6.17	42.96 ± 5.50	28.34 ± 4.17
PC	—	7.19 ± 1.06	5.72 ± 1.64	4.96 ± 1.24	8.54 ± 0.97
PS + PI	—	2.80 ± 2.09	6.27 ± 2.35	3.18 ± 1.80	5.09 ± 1.45
PG	—	5.12 ± 0.70	9.86 ± 1.79	9.96 ± 2.23	12.91 ± 1.96
PE	—	8.79 ± 1.59	9.32 ± 1.47	8.96 ± 2.87	13.97 ± 1.95
Polar lipid	—	23.91 ± 5.42	31.17 ± 6.97	27.06 ± 4.92	40.51 ± 6.09
Neutral lipid	—	71.97 ± 7.95	64.39 ± 6.89	72.94 ± 4.92	59.49 ± 6.09
unknown		4.11 ± 2.53	4.43 ± 0.21	1.41 ± 2.45	12.30 ± 1.69

^*^Significant differences (*P* ≤ 0.05) between cultures given by Student's *t*-test when comparing the mean of percentages of the lipid classes for cultures B1 and C1.

^a,b^Significant differences of TL values, also showing homogeneous subclusters as indicated by Tuckey's test when comparing the mean of TL values.

TL: total lipid; PC: phosphatidylcholine; PS + PI: sum of phosphatidylserine and phosphatidylinositol; PG: phosphatidylglycerol; PE: phosphatidylethanolamine.

Percentage values were transformed by the arcsin transformation prior to statistical analysis.

Values represent the mean of three independent experiments (*n* = 3) ± SD.

Entry of culture B1 for sampling point 5 is empty because data were collected only for sampling 4.

**Table 3:** Principal components (PC1, PC2) of the studied lipid classes in *Echium acanthocarpum* E1.5 cell line hairy roots, growing in different culture media at 15°C (C1–C4). Factor loadings and communalities are shown.

Components matrix	Components		Communalities
	PC1 (65.91%)	PC2 (19.78%)	Extraction
PC	**0.886**	−0.339	0.900
PS + PI	**0.759**	0.094	0.585
PG	0.033	*0.966 *	0.934
PE	**0.767**	0.382	0.735
Polar lipid	**0.846**	0.510	0.975
Neutral lipid	**−0.715**	−0.582	0.850

Factor loading of the variable (correlations) with PC1 are shown in bold, and those correlated with PC2 are shown in italic.

PC: phosphatidylcholine; PS + PI: phosphatidylserine and phosphatidylinositol; PG: phosphatidylglycerol; PE: phosphatidylethanolamine.

Rotation method: Varimax with Kaiser.

**Table 4:** Two-way ANOVA (stress conditions and sampling time) of the two principal components PC1 and PC2 of recorded lipid classes in *Echium acanthocarpum* E1.5 cell line hairy root cultures (C1–C4), growing at 15°C.

Two-way ANOVA	Stress conditions(osmotic pressure and carbon source)		Time (sampling point)		Interaction between factors	
	*F*-value	Sign.	*F*-value	Sign.	*F*-value	Sign.
PC1	0.167	0.917	3.379	0.085	3.474	0.041
PC2	9.537	0.001	1.815	0.197	2.434	0.103

Sign.: significance (*P* ≤ 0.05).

**Table 5:** Total lipid content (TL; mg/g DW), total fatty acid content (FA, mg/g DW), and the percentage of each fatty acid (%) of *Echium acanthocarpum* hairy roots at sampling point 4 cultured for 20 days (culture B1) or 45 days (cultures C1–C4).

	Sampling point 4				
	B1	C1	C2	C3	C4
TL (mg/g DW)	33.41 ± 3.66	41.67 ± 2.45	35.05 ± 4.50	32.20 ± 4.94	31.03 ± 1.03
FA (mg/g DW)	7.30 ± 6.28	18.90 ± 3.85	9.74 ± 0.55	12.80 ± 4.00	8.92 ± 1.26
Fatty acids					
14:0	0.22 ± 0.02	0.15 ± 0.01	ND	0.10 ± 0.09	0.12 ± 0.11
16:0	26.53 ± 1.73	20.03 ± 0.99	23.43 ± 2.02	23.02 ± 2.40	24.49 ± 1.10
18:0	2.92 ± 0.30	1.48 ± 0.06	1.94 ± 0.24	1.88 ± 0.30	2.20 ± 0.22
18:1n-9	4.56 ± 0.92	8.29 ± 1.70	5.69 ± 0.71	6.26 ± 1.09	4.94 ± 1.07
18:1n-7	1.53 ± 0.05	0.60 ± 0.04	0.87 ± 1.19	0.90 ± 0.16	1.10 ± 0.07
18:2n-6 (LA)	36.82 ± 0.99	35.79 ± 0.65	33.98 ± 2.00	35.71 ± 1.09	36.70 ± 1.41
18:3n-6 (GLA)	13.88 ± 0.78	18.37 ± 0.70	18.99 ± 2.51	18.15 ± 1.51	17.69 ± 1.53
18:3n-3 (ALA)	4.24 ± 0.20	5.16 ± 0.65	6.71 ± 0.80	6.17 ± 0.56	6.16 ± 0.54
18:4n-3 (SDA)	1.26 ± 0.45	1.96 ± 0.47	3.06 ± 0.67	2.77 ± 0.43	1.29 ± 0.89
20:0	0.29 ± 0.06	0.45 ± 0.04	0.60 ± 0.03	0.53 ± 0.08	0.57 ± 0.10
22:0	2.45 ± 0.31	1.56 ± 0.12	1.71 ± 0.16	1.60 ± 0.04	1.55 ± 0.18
24:0	1.94 ± 0.49	0.18 ± 0.02	1.28 ± 0.07	1.37 ± 0.12	0.29 ± 0.06
Unknown	2.36 ± 0.35	2.63 ± 0.71	1.26 ± 0.25	1.25 ± 0.32	1.50 ± 0.06

GLA + SDA	15.14 ± 1.23	20.33 ± 1.17	22.05 ± 3.18	20.92 ± 1.94	18.98 ± 2.42
Total saturated FA	34.36 ± 1.67	25.36 ± 0.92	28.97 ± 2.09	28.50 ± 2.54	30.33 ± 1.08
Total monoene FA	7.07 ± 0.73	10.72 ± 1.90	7.03 ± 0.70	7.45 ± 0.90	6.33 ± 1.15
n-9	5.33 ± 0.66	9.94 ± 1.95	5.69 ± 0.71	6.26 ± 1.09	4.94 ± 1.07
n-6	50.70 ± 1.66	54.16 ± 0.95	52.97 ± 0.54	53.85 ± 1.51	54.39 ± 2.87
n-3	5.50 ± 0.53	7.12 ± 1.10	9.76 ± 1.46	8.94 ± 0.94	7.45 ± 0.84
n-3/n-6	0.11 ± 0.01	0.13 ± 0.02	0.18 ± 0.03	0.17 ± 0.02	0.14 ± 0.02
Δ6-des index (n-6)	0.27 ± 0.01	0.34 ± 0.01	0.36 ± 0.04	0.34 ± 0.02	0.32 ± 0.01
Δ6-des index (n-3)	0.23 ± 0.06	0.27 ± 0.02	0.31 ± 0.02	0.31 ± 0.02	0.17 ± 0.10
DBI	1.40 ± 0.06	1.61 ± 0.03	1.64 ± 0.09	1.63 ± 0.07	1.56 ± 0.03

ND: Not detected. n-6 and n-3 Δ6-desaturation indexes were calculated as 18:3n-6/(18:3n-6 + 18:2n-6) and 18:4n-3/(18:4n-3 + 18:3n-3), respectively.

Double bond index (DBI) was calculated as [(%18:1n) + 2 × (%18:2n) + 3 × (%18:3n) + 4 × (18:4n)]/100. Values represent the mean of three replicates (*n* = 3) ± SD.

**Table 6:** Total lipid content (TL; mg/g DW), total fatty acids content (FA; mg/g DW), and percentages of each fatty acid of *Echium acanthocarpum* hairy roots at sampling point 5 cultured for 25 days (culture B1) or 55 days (cultures C1–C4).

	Sampling point 5				
	B1	C1	C2	C3	C4
TL (mg/g DW)	35.00 ± 5.40	35.58 ± 7.33	28.59 ± 6.17	42.96 ± 5.50	28.34 ± 4.17
FA (mg/g DW)	8.45 ± 0.87	13.71 ± 4.10	9.38 ± 0.84	13.29 ± 3.43	9.90 ± 1.71
Fatty acids					
14:0	0.23 ± 0.02	0.14 ± 0.03	0.06 ± 0.10	0.08 ± 0.14	0.11 ± 0.09
16:0	25.04 ± 1.14	20.21 ± 1.81	22.02 ± 0.33	21.66 ± 0.21	23.38 ± 0.70
18:0	2.55 ± 0.21	1.82 ± 0.48	1.91 ± 0.24	1.93 ± 0.31	1.86 ± 0.15
18:1n-9	6.77 ± 1.10	8.79 ± 2.43	4.82 ± 1.14	5.66 ± 0.49	4.24 ± 1.65
18:1n-7	1.55 ± 0.16	0.70 ± 0.06	0.74 ± 0.09	0.86 ± 0.26	0.97 ± 0.14
18:2n-6 (LA)	34.55 ± 1.97	34.34 ± 3.77	34.14 ± 3.44	31.95 ± 2.95	38.08 ± 1.47
18:3n-6 (GLA)	11.77 ± 1.63	17.51 ± 1.77	19.79 ± 0.61	19.92 ± 0.98	19.23 ± 0.27
18:3n-3 (ALA)	4.22 ± 0.34	5.32 ± 0.56	5.85 ± 1.60	5.96 ± 1.17	6.22 ± 0.40
18:4n-3 (SDA)	0.88 ± 0.21	2.12 ± 0.19	2.73 ± 0.47	2.75 ± 0.43	1.27 ± 0.55
20:0	0.27 ± 0.04	0.64 ± 0.30	0.84 ± 0.25	0.80 ± 0.38	0.42 ± 0.05
22:0	2.51 ± 0.35	2.42 ± 1.50	2.52 ± 1.84	2.83 ± 1.32	1.35 ± 0.09
24:0	2.08 ± 0.60	0.21 ± 0.02	0.40 ± 0.17	1.81 ± 0.45	0.21 ± 0.03
Unknown	6.08 ± 2.68	2.23 ± 0.46	1.60 ± 1.12	3.50 ± 1.64	1.23 ± 0.53

GLA + SDA	12.65 ± 1.84	19.63 ± 1.96	22.52 ± 1.08	22.67 ± 1.43	20.50 ± 0.82
Total saturated FA	32.68 ± 2.21	27.15 ± 4.25	29.37 ± 2.84	29.11 ± 2.58	28.55 ± 0.60
Total monoene FA	9.54 ± 1.29	11.32 ± 2.42	6.52 ± 1.91	6.81 ± 0.61	5.42 ± 1.81
n-9	7.59 ± 1.15	10.40 ± 2.41	5.38 ± 2.04	5.66 ± 0.49	4.24 ± 1.65
n-6	46.32 ± 3.60	51.85 ± 2.00	53.93 ± 3.67	51.87 ± 2.31	57.31 ± 1.70
n-3	5.09 ± 0.53	7.44 ± 0.42	8.58 ± 2.07	8.71 ± 1.54	7.49 ± 0.16
n-3/n-6	0.11 ± 0.01	0.14 ± 0.01	0.16 ± 0.03	0.17 ± 0.00	0.13 ± 0.00
Δ6-des index (n-6)	0.25 ± 0.02	0.34 ± 0.05	0.37 ± 0.02	0.38 ± 0.02	0.34 ± 0.01
Δ6-des index (n-3)	0.17 ± 0.02	0.29 ± 0.04	0.32 ± 0.02	0.32 ± 0.03	0.17 ± 0.07
DBI	1.30 ± 0.08	1.57 ± 0.05	1.63 ± 0.12	1.59 ± 0.10	1.63 ± 0.03

n-6 and n-3 Δ6-desaturation indexes were calculated as 18:3n-6/(18:3n-6 + 18:2n-6) and 18:4n-3/(18:4n-3 + 18:3n-3), respectively. Double Bond Index (DBI) was calculated as [(%18:1n) + 2 × (%18:2n)  + 3 × (%18:3n) + 4 × (18:4n)]/100. Values represent the mean of three replicates (*n* = 3) ± SD.

**Table 7:** Principal components (PC1 y PC2) of fatty acids studied in *Echium acanthocarpum* hairy roots, growing at different temperatures (25 and 15°C) in culture media (B1, C1).

Matrix components	Components		Communalities
	PC1 (48.95%)	PC2 (17.92%)	Extraction
14:0	**0.767**	0.265	0.658
16:0	**0.929**	0.112	0.876
18:0	**0.896**	0.163	0.829
18:1n-9	**−0.550**	0.119	0.317
18:1n-7	**0.949**	−0.091	0.909
18:2n-6	0.170	−*0.826 *	0.710
18:3n-6	**−0.888**	−0.021	0.788
18:3n-3	**−**0.335	−*0.530 *	0.394
18:4n-3	**−0.561**	0.441	0.509
20:0	**−0.658**	0.565	0.752
22:0	0.240	*0.730 *	0.591
24:0	**0.897**	0.097	0.813

bold values show factor loadings of variables (correlations) with PC1 and in italic the correlations with PC2.

Rotation method: Varimax with Kaiser normalization.

**Table 8:** Two-way ANOVA test (stress factor and time) of PC1 and PC2 of detected fatty acids from *Echium acanthocarpum* hairy roots, grown at different temperature 25°C and 15°C (cultures B1, C1).

Two-way ANOVA	Stress factor (Temperature)		Time (Sampling point)		Interaction between factors	
	*F*-value	Sign.	*F*-value	Sign.	*F*-value	Sign.
PC1	214.056	0.000	7.734	0.000	2.174	0.091
PC2	1.126	0.299	0.464	0.799	3.162	0.025

PC: principal component; Sign.: significance (*P* ≤ 0.05).

**Table 9:** Principal components (PC1 y PC2) of fatty acid profiles studied in *Echium acanthocarpum* hairy roots, growing in different culture media (C1–C4).

Matrix Components	Components		Communalities
	PC1 (30.772%)	PC2 (21.729%)	Extraction
14:0	−0.174	−*0.233 *	0.085
16:0	**0.769**	0.116	0.604
18:0	**0.876**	0.074	0.772
18:1n-9	−0.328	−0.029	0.109
18:1n-7	**0.838**	−0.268	0.775
18:2n-6	0.313	−*0.723 *	0.620
18:3n-6	**−0.767**	0.125	0.604
18:3n-3	0.105	−*0.538 *	0.300
18:4n-3	**−0.704**	0.108	0.507
20:0	0.175	*0.899 *	0.839
22:0	0.038	*0.926 *	0.860
24:0	−0.316	*0.355 *	0.226

Bold values show factor loadings of variables (correlations) with PC1 and in italic the factor loadings the correlations with PC2.

Rotation method: Varimax with Kaiser normalization.

**Table 10:** Two-way ANOVA (stress conditions and sampling time) of PC1 and Trans-PC2 of fatty acid profiles in* Echium acanthocarpum* hairy roots, growing in different culture media at 15°C (cultures C1–C4).

Two-way ANOVA	Stress conditions(osmotic pressure and carbon source)		Time (sampling point)		Interaction between factors	
	*F*-value	Sign.	*F*-value	Sign.	*F*-value	Sign.
PC1	13.912	0.000	24.725	0.000	8.315	0.000
Trans-PC2	9.534	0.000	2.882	0.024	1.610	0.106

PC: principal component; Trans-PC2: transformed principal component 2 (NL (PC2-min (PC2) + 1)); Sign.: significance (*P* ≤ 0.05).
