# Supplementary material for: Improvement of Polyunsaturated Fatty Acid Production in Echium acanthocarpum Transformed Hairy Root Cultures by Application of Different Abiotic Stress Conditions
Source: ISRN Biotechnol. 2013 Nov 13;2013:169510. doi: 10.5402/2013/169510 (PMC4393039; doi:10.5402/2013/169510)
Supplement: Supplementary file 1 — The additional information accessible on-line provides data presented in tables and figures of the different parameters recorded in this investigation at the other sampling times of the different culture growth curves, as well as additional data of the statistical analyses conducted and not present in the printed version of the publication. [file 169510.f1.pdf]

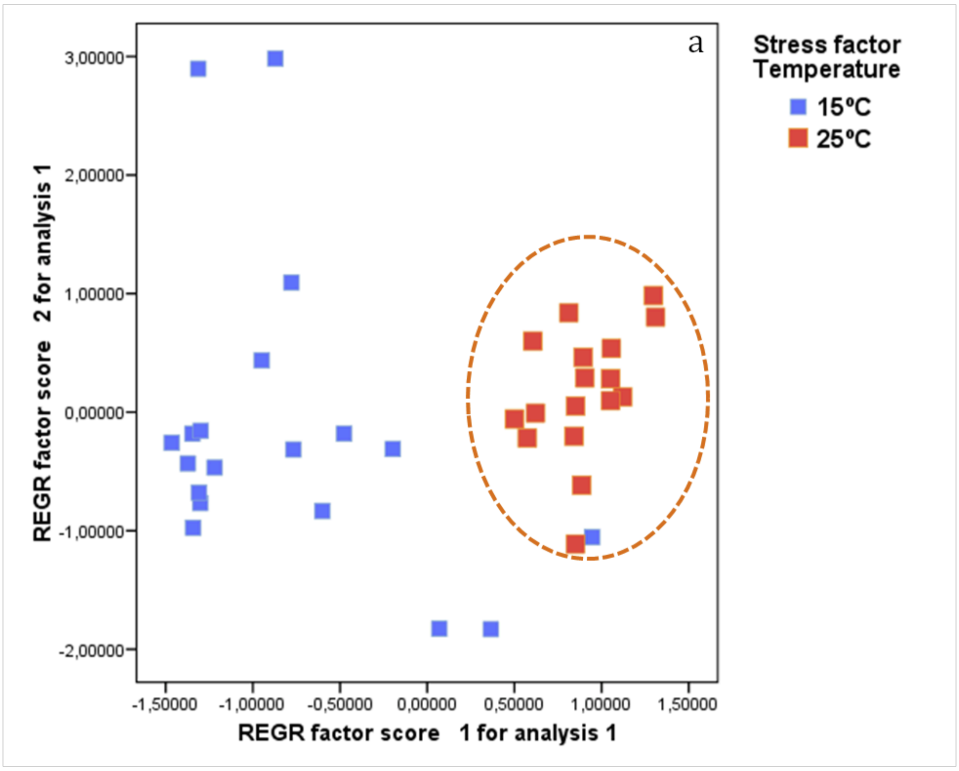

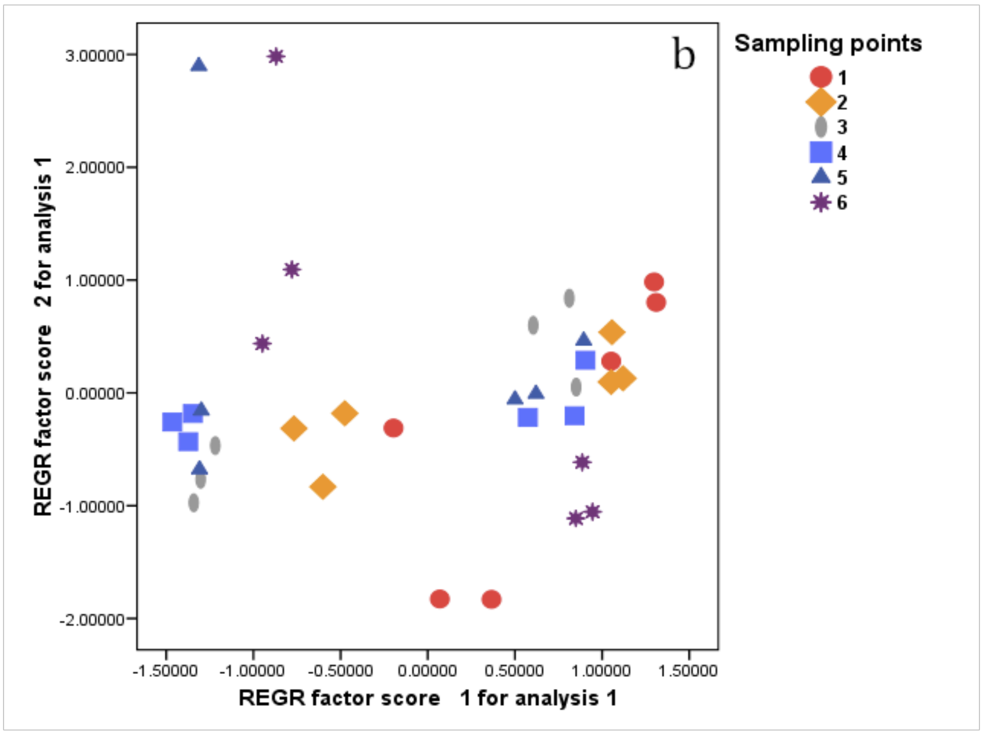

Figures S1a,b. Plot of each hairy root sample according to PC1 and PC2 stratified by the stress factor temperature (a) or sampling points (b).

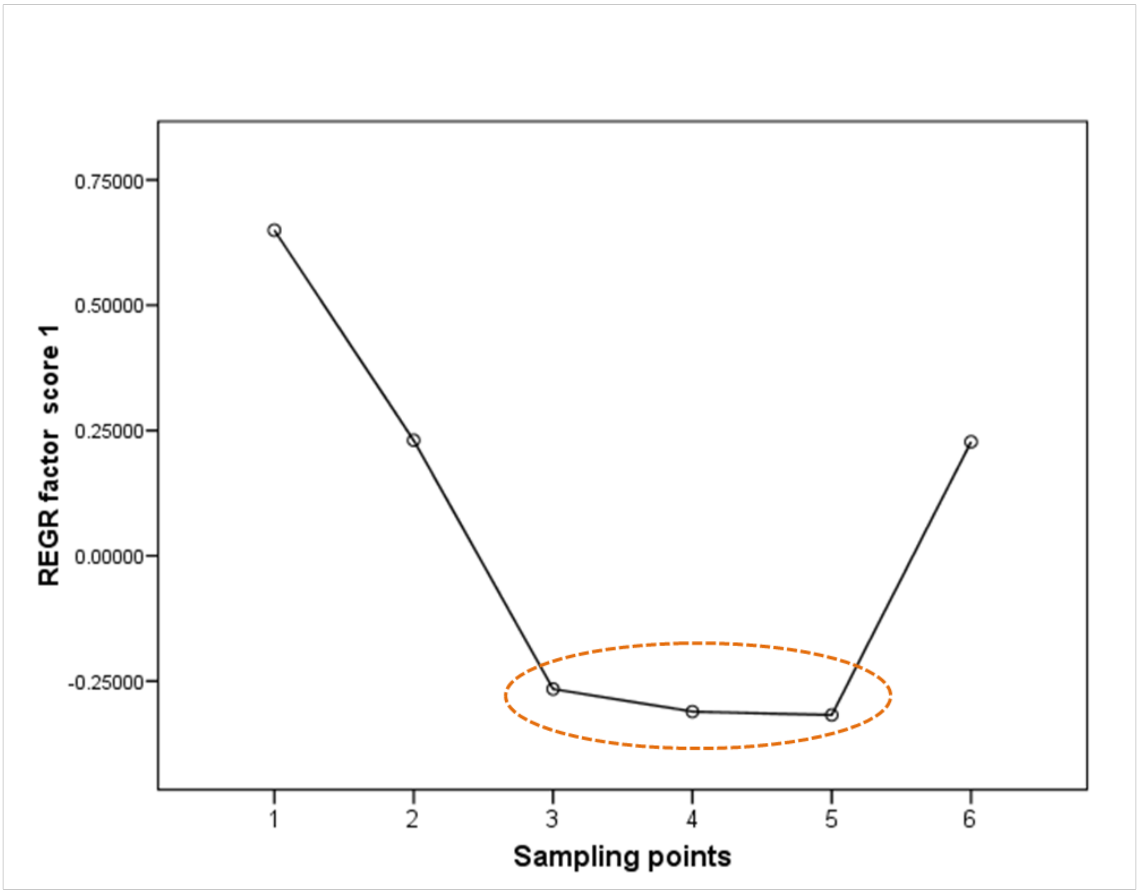

Figure S2. Plot of the estimated marginal means of PC1 against sampling points

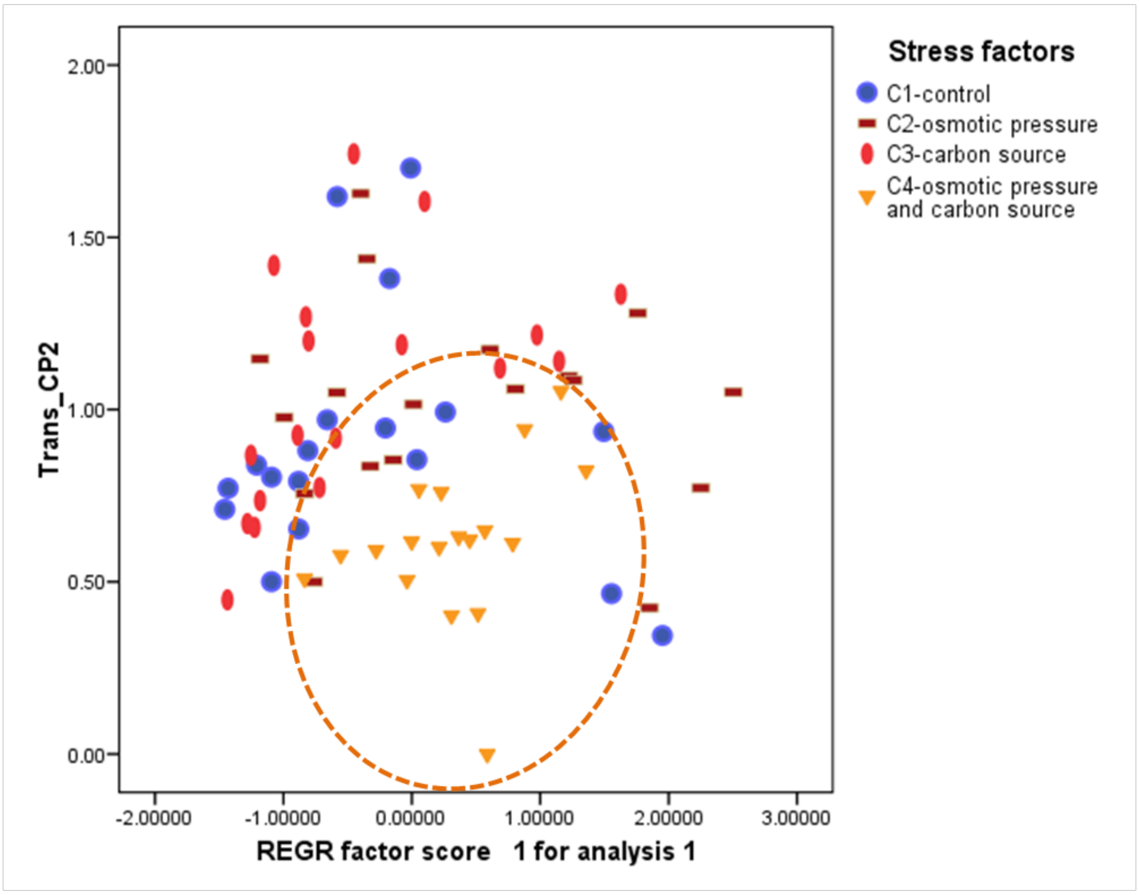

Figure S3. Plot of the principal components stratified according to the stress factor present in each culture (cultures C1-C4).

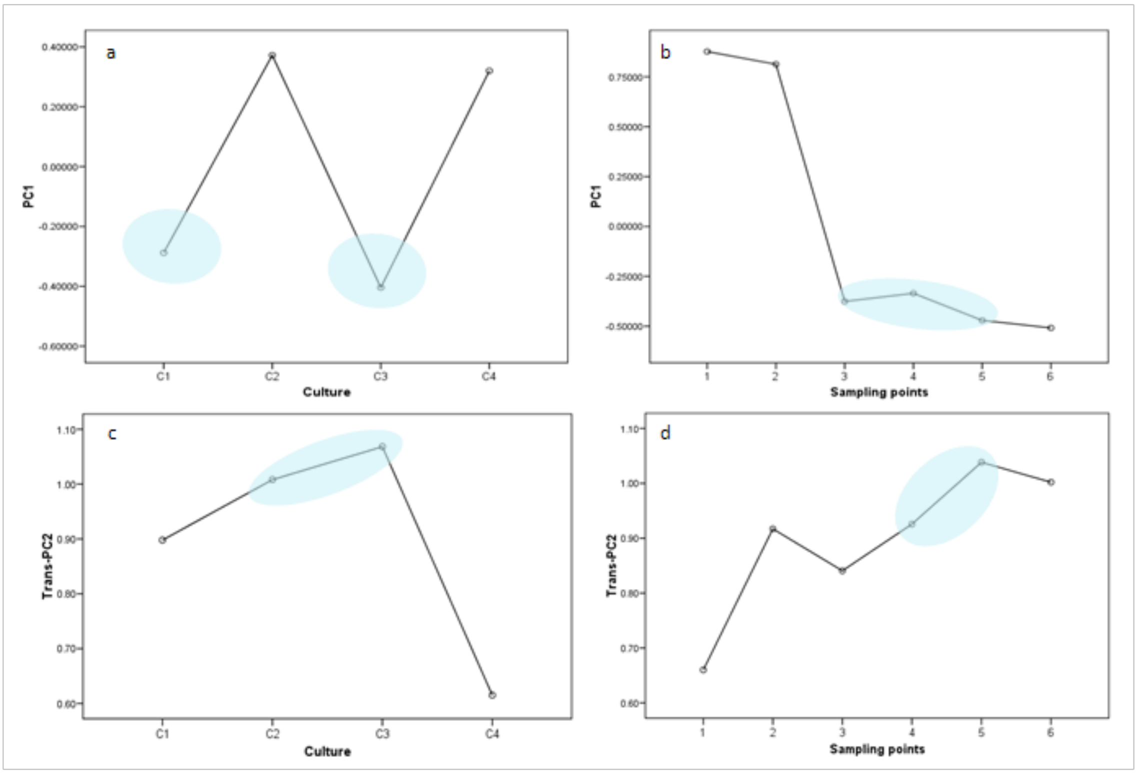

Figure S4. Plot of the PC1 estimated marginal means as a function of (a) culture medium and (b) sampling points. Plot of the PC2 estimated marginal means as a function of (c) culture medium and (d) sampling points. Blue areas indicate the most interesting values of these components according to each studied factor.

Table S1. Total lipid content (TL; mg/g DW), total fatty acids content (FAs; mg/g DW), and percentages of each fatty acid of *Echium acanthocarpum* hairy roots at sampling point 1 cultured for 5 days (culture B1) or 15 days (cultures C1-C4). n-6 and n-3 Δ6-desaturation indexes were calculated as 18:3n-6/(18:3n-6+18:2n-6) and 18:4n-3/(18:4n-3+18:3n-3), respectively. Double Bond Index (DBI) was calculated as [(%18:1n)+2x(%18:2n)+3x(%18:3n)+4x(18:4n)]/100. Values represent the mean of three replicates (n=3) ± SD.

	Sampling point 1				
	B1	*C1*	*C2*	*C3*	*C4*
TL (mg/g DW)	60.95 ± 6.10	48.13 ± 3.85	51.29 ± 3.61	47.27 ± 6.13	56.05 ± 2.72
FA (mg/g DW)	9.18 ± 1.10	10.28 ± 1.13	10.40 ± 1.97	18.12 ± 2.90	18.12 ± 3.14
Fatty acids					
14:0	0.83 ± 0.23	0.26 ± 0.04	*ND*	0.13 ± 0.02	0.36 ± 0.16
16:0	26.64 ± 1.03	23.03 ± 0.73	24.15 ± 0.78	21.29 ± 0.25	23.65 ± 1.71
18:0	3.78 ± 0.20	2.61 ± 0.15	3.11 ± 0.22	1.88 ± 0.18	2.80 ± 0.54
18:1n-9	6.29 ± 1.13	5.92 ± 0.11	6.29 ± 0.73	5.76 ± 0.43	5.48 ± 0.82
18:1 n-7	2.05 ± 0.17	1.18 ± 0.44	1.33 ± 0.10	0.79 ± 0.10	1.17 ± 0.17
18:2n-6 (LA)	33.57 ± 1.42	38.11 ± 3.06	39.10 ± 2.53	34.81 ± 0.44	37.86 ± 3.19
18:3n-6 (GLA)	10.46 ± 0.86	14.90 ± 3.38	12.72 ± 0.27	20.13 ± 0.91	15.53 ± 3.54
18:3n-3 (ALA)	5.13 ± 0.28	6.25 ± 0.60	7.33 ± 0.62	8.30 ± 0.28	6.81 ± 1.63
18:4n-3 (SDA)	1.33 ± 0.11	0.38 ± 0.34	0.77 ± 0.12	3.45 ± 0.49	1.79 ± 1.34
20:0	0.34 ± 0.01	0.47 ± 0.17	0.68 ± 0.11	0.45 ± 0.07	0.55 ± 0.07
22:0	2.11 ± 0.06	1.68 ± 0.09	1.63 ± 0.53	1.26 ± 0.20	1.46 ± 0.33
24:0	1.58 ± 0.12	1.35 ± 0.26	1.57 ± 0.33	0.82 ± 0.12	1.08 ± 0.36
Unknown	4.88 ± 0.12	2.65 ± 0.37	0.95 ± 0.96	0.75 ± 0.44	1.25 ± 0.38
GLA + SDA	11.79 ± 0.97	15.28 ± 3.72	13.49 ± 0.39	23.58 ± 1.40	19.07 ± 4.88
Total Saturated FA	35.28 ± 1.50	29.41 ± 0.62	31.13 ± 1.87	25.82 ± 0.72	29.90 ± 2.85
Total Monoene FA	8.89 ± 0.71	8.31 ± 0.46	7.99 ± 0.86	6.74 ± 0.49	6.87 ± 0.87
n-9	6.44 ± 0.53	6.92 ± 0.11	6.66 ± 0.79	5.76 ± 0.43	5.48 ± 0.82
n-6	44.03 ± 2.18	53.00 ± 0.59	51.82 ± 2.55	54.94 ± 1.17	53.39 ± 1.60
n-3	6.45 ± 0.31	6.62 ± 0.94	8.10 ± 0.64	11.75 ± 0.28	8.59 ± 2.79
n-3/n-6	0.15 ± 0.01	0.12 ± 0.02	0.16 ± 0.01	0.21 ± 0.00	0.16 ± 0.05
Δ6-des index (n-6)	0.24 ± 0.01	0.28 ± 0.06	0.25 ± 0.01	0.37 ± 0.01	0.29 ± 0.06
Δ6-des index(n-3)	0.21 ± 0.01	0.05 ± 0.04	0.10 ± 0.02	0.29 ± 0.04	0.19 ±0.10
DBI	1.29 ± 0.05	1.49 ± 0.07	1.49 ± 0.05	1.75 ± 0.04	1.57 ± 0.14

Table S2. Total lipid content (TL; mg/g DW), total fatty acids content (FAs; mg/g DW), and percentages of each fatty acid of Echium acanthocarpum hairy roots at sampling point 1 cultured for 10 days (culture B1) or 25 days (cultures C1-C4). n-6 and n-3 Δ6-desaturation indexes were calculated as 18:3n-6/(18:3n-6+18:2n-6) and 18:4n-3/(18:4n-3+18:3n-3), respectively. Double Bond Index (DBI) was calculated as [(%18:1n)+2x(%18:2n)+3x(%18:3n)+4x(18:4n)]/100. Values represent the mean of three replicates (n=3) ± SD.

	Sampling point 2				
	B1	*C1*	*C2*	*C3*	*C4*
TL (mg/g DW)	40.74 ± 1.92	42.38 ± 8.98	47.12 ± 6.26	35.85 ± 7.55	33.04 ± 4.75
FA (mg/g DW)	7.43 ± 0.98	13.06 ± 2.14	10.79 ± 1.40	8.15 ± 1.08	10.13 ± 1.72
Fatty acids					
14:0	0.49 ± 0.12	0.15 ± 0.01	*ND*	0.03 ± 0.07	0.26 ± 0.06
16:0	26.84 ± 0.79	23.58 ± 0.17	24.58 ± 1.26	24.30 ± 1.18	22.96 ± 0.17
18:0	3.08 ± 0.16	1.83 ± 0.16	2.62 ± 0.57	2.44 ± 0.59	2.72 ± 0.31
18:1n-9	5.25 ± 0.73	5.12 ± 0.86	4.70 ± 0.97	4.91 ± 0.90	4.42 ± 0.85
18:1 n-7	1.76 ± 0.05	0.98 ± 0.23	1.25 ± 0.17	1.12 ± 0.31	1.12 ± 0.09
18:2n-6 (LA)	36.14 ± 1.15	34.86 ± 1.46	34.66 ± 2.00	34.22 ± 1.85	37.98 ± 0.97
18:3n-6 (GLA)	12.05 ± 0.41	18.97 ± 0.33	16.56 ± 1.63	17.58 ± 2.44	17.45 ± 0.63
18:3n-3 (ALA)	4.42 ± 0.22	5.64 ± 0.35	6.83 ± 1.15	6.70 ± 0.97	7.75 ± 0.55
18:4n-3 (SDA)	1.15 ± 0.10	0.99 ± 0.28	1.62 ± 0.59	1.93 ± 0.79	2.06 ± 0.42
20:0	0.41 ± 0.13	0.47 ± 0.01	0.67 ± 0.14	0.67 ± 0.11	0.48 ± 0.07
22:0	2.06 ± 0.12	1.72 ± 0.18	2.15 ± 0.33	2.08 ± 0.30	1.04 ± 0.37
24:0	1.56 ± 0.04	1.47 ± 0.16	1.35 ± 0.12	1.28 ± 0.16	0.65 ± 0.34
Unknown	3.86 ± 0.62	2.79 ± 0.41	2.73 ± 1.66	2.74 ± 0.44	0.88 ± 0.21
GLA + SDA	13.2 ± 0.51	19.96 ± 0.61	18.17 ± 2.22	19.51 ± 3.23	19.46 ± 1.05
Total Saturated FA	34.44 ± 0.50	29.21 ± 0.21	31.37 ± 1.81	30.80 ± 1.87	28.10 ± 0.57
Total Monoene FA	7.93 ± 0.91	7.54 ± 1.35	6.23 ± 1.18	6.31 ± 0.98	5.77 ± 0.91
n-9	5.81 ± 0.83	6.05 ± 0.83	4.70 ± 0.97	4.91 ± 0.90	4.42 ± 0.85
n-6	48.20 ± 1.52	53.83 ± 1.75	51.22 ± 0.69	51.80 ± 1.30	55.43 ± 1.19
n-3	5.57 ± 0.26	6.63 ± 0.34	8.45 ± 1.52	8.63 ± 1.29	9.81 ± 0.86
n-3/n-6	0.12 ± 0.00	0.12 ± 0.01	0.17 ± 0.03	0.17 ± 0.03	0.18 ± 0.02
Δ6-des index (n-6)	0.25 ± 0.00	0.35 ± 0.01	0.32 ± 0.03	0.34 ± 0.04	0.31 ± 0.01
Δ6-des index(n-3)	0.21 ± 0.02	0.15 ± 0.04	0.19 ± 0.05	0.22 ± 0.08	0.21 ± 0.03
DBI	1.34 ± 0.05	1.55 ± 0.01	1.52 ± 0.05	1.55 ± 0.07	1.66 ± 0.01

Table S3. Total lipid content (TL; mg/g DW), total fatty acids content (FAs; mg/g DW), and percentages of each fatty acid of Echium acanthocarpum hairy roots at sampling point 1 cultured for 15 days (culture B1) or 35 days (cultures C1-C4). n-6 and n-3 Δ6-desaturation indexes were calculated as 18:3n-6/(18:3n-6+18:2n-6) and 18:4n-3/(18:4n-3+18:3n-3), respectively. Double Bond Index (DBI) was calculated as [(%18:1n)+2x(%18:2n)+3x(%18:3n)+4x(18:4n)]/100. Values represent the mean of three replicates (n=3) ± SD.

	Sampling point 3				
	B1	*C1*	*C2*	*C3*	*C4*
TL (mg/g DW)	38.13 ± 5.35	40.57 ± 13.91	40.40 ± 8.55	36.46 ± 4.63	33.00 ± 4.45
FA (mg/g DW)	7.45 ± 1.63	13.88 ± 2.30	8.40 ± 1.76	14.05 ± 2.29	10.71 ± 1.39
Fatty acids					
14:0	0.39 ± 0.04	0.14 ± 0.03	0.14 ± 0.24	0.15 ± 0.04	0.14 ± 0.03
16:0	26.19 ± 0.61	20.84 ± 0.32	23.50 ± 1.42	23.10 ± 1.65	22.45 ± 0.57
18:0	2.98 ± 0.12	1.61 ± 0.31	2.16 ± 0.18	1.86 ± 0.20	2.20 ± 0.29
18:1n-9	6.38 ± 0.57	6.03 ± 0.30	4.79 ± 0.86	6.48 ± 1.17	4.29 ± 1.17
18:1 n-7	1.74 ± 0.07	0.64 ± 0.12	1.01 ± 0.03	0.96 ± 0.40	0.97 ± 0.16
18:2n-6 (LA)	36.10 ± 1.39	36.61 ± 0.23	34.46 ± 0.57	35.00 ± 1.93	38.01 ± 0.16
18:3n-6 (GLA)	13.21 ± 0.67	19.23 ± 0.39	18.36 ± 1.27	18.46 ± 2.15	18.68 ± 1.56
18:3n-3 (ALA)	4.11 ± 0.40	6.18 ± 0.40	6.99 ± 0.87	6.17 ± 0.44	6.97 ± 0.60
18:4n-3 (SDA)	1.12 ± 0.03	1.85 ± 0.64	2.93 ± 0.52	2.71 ± 0.32	1.78 ± 0.10
20:0	0.39 ± 0.03	0.50 ± 0.07	0.60 ± 0.09	0.60 ± 0.08	0.46 ± 0.04
22:0	2.39 ± 0.15	1.54 ± 0.22	1.55 ± 0.15	1.67 ± 0.42	1.43 ± 0.26
24:0	1.40 ± 0.10	0.17 ± 0.01	0.43 ± 0.09	1.11 ± 0.49	0.26 ± 0.02
Unknown	2.66 ± 0.07	2.09 ± 0.28	1.74 ± 0.66	1.38 ± 0.12	1.03 ± 0.12
GLA + SDA	14.33 ± 0.70	21.08 ± 1.03	21.29 ± 1.79	21.17 ± 2.47	20.46 ± 1.66
Total Saturated FA	33.73 ± 0.79	25.88 ± 0.80	29.29 ± 2.05	28.50 ± 2.86	28.01 ± 0.97
Total Monoene FA	9.07 ± 0.59	8.15 ± 0.56	6.23 ± 0.83	7.79 ± 1.16	5.53 ± 1.32
n-9	7.00 ± 0.57	7.34 ± 0.56	4.79 ± 0.86	6.48 ± 1.17	4.29 ± 1.17
n-6	49.31 ± 1.00	55.85 ± 0.30	52.81 ± 1.84	53.45 ± 1.69	56.69 ± 1.41
n-3	5.23 ± 0.39	8.04 ± 1.04	9.93 ± 1.39	8.87 ± 0.76	8.74 ± 0.56
n-3/n-6	0.11 ± 0.01	0.14 ± 0.02	0.19 ± 0.02	0.17 ± 0.01	0.15 ± 0.01
Δ6-des index (n-6)	0.27 ± 0.02	0.34 ± 0.01	0.35 ± 0.01	0.35 ± 0.04	0.33 ± 0.02
Δ6-des index(n-3)	0.21 ± 0.02	0.23 ± 0.05	0.29 ± 0.01	0.30 ± 0.01	0.20 ± 0.02
DBI	1.38 ± 0.02	1.65 ± 0.03	1.63 ± 0.09	1.62 ± 0.08	1.66 ± 0.03

Table S4. Total lipid content (TL; mg/g DW), total fatty acids content (FAs; mg/g DW), and percentages of each fatty acid of Echium acanthocarpum hairy roots at sampling point 1 cultured for 35 days (culture B1) or 75 days (cultures C1-C4). n-6 and n-3 Δ6-desaturation indexes were calculated as 18:3n-6/(18:3n-6+18:2n-6) and 18:4n-3/(18:4n-3+18:3n-3), respectively. Double Bond Index (DBI) was calculated as [(%18:1n)+2x(%18:2n)+3x(%18:3n)+4x(18:4n)]/100. Values represent the mean of three replicates (n=3) ± SD.

	Sampling point 6				
	B1	*C1*	*C2*	*C3*	*C4*
TL (mg/g DW)	31.38 ± 1.48	33.41 ± 11.54	25.66 ± 5.12	30.61 ± 4.11	35.56 ± 5.13
FA (mg/g DW)	7.44 ± 0.99	11.54 ± 6.99	7.37 ± 0.83	8.85 ± 3.79	10.30 ± 1.60
Fatty acids					
14:0	0.19 ± 0.01	0.18 ± 0.04	0.05 ± 0.08	*ND*	0.15 ± 0.02
16:0	25.39 ± 0.54	21.01 ± 0.81	21.76 ± 0.70	20.78 ± 1.51	21.66 ± 1.28
18:0	2.57 ± 0.12	1.78 ± 0.25	1.62 ± 0.11	1.75 ± 0.22	1.81 ± 0.29
18:1n-9	4.14 ± 0.54	7.29 ± 3.54	5.23 ± 0.40	5.95 ± 0.93	5.79 ± 0.78
18:1 n-7	2.30 ± 0.21	0.74 ± 0.16	0.83 ± 0.06	0.85 ± 0.09	0.92 ± 0.09
18:2n-6 (LA)	38.13 ± 0.52	34.00 ± 3.33	36.62 ± 1.52	35.93 ± 1.16	37.30 ± 1.69
18:3n-6 (GLA)	14.17 ± 0.85	16.78 ± 0.82	19.00 ± 1.30	18.30 ± 0.90	18.10 ± 0.55
18:3n-3 (ALA)	5.27 ± 0.21	4.56 ± 1.53	6.94 ± 0.35	5.87 ± 1.60	7.03 ± 0.72
18:4n-3 (SDA)	0.76 ± 0.21	1.62 ± 0.60	2.89 ± 0.39	2.37 ± 0.59	2.53 ± 0.50
20:0	0.19 ± 0.01	0.78 ± 0.32	0.58 ± 0.03	0.72 ± 0.28	0.52 ± 0.03
22:0	2.62 ± 0.22	3.40 ± 1.78	1.70 ± 0.14	2.54 ± 1.13	1.41 ± 0.09
24:0	1.75 ± 0.07	2.70 ± 1.06	1.52 ± 0.28	2.22 ± 0.86	1.32 ± 0.20
Unknown	1.77 ± 0.01	2.75 ± 0.82	0.99 ± 0.01	2.44 ± 1.02	1.26 ± 0.21
GLA + SDA	14.93 ± 1.06	18.40 ± 1.42	21.89 ± 1.69	20.67 ± 1.49	20.63 ± 1.05
Total Saturated FA	32.71 ± 0.52	29.84 ± 4.19	27.23 ± 0.92	28.01 ± 3.95	26.88 ± 1.50
Total Monoene FA	7.20 ± 0.60	10.45 ± 3.36	6.32 ± 0.29	7.08 ± 0.98	6.90 ± 0.68
n-9	4.82 ± 0.46	9.50 ± 3.23	5.23 ± 0.40	5.95 ± 0.93	5.79 ± 0.78
n-6	52.29 ± 1.15	50.78 ± 2.70	55.62 ± 0.35	54.23 ± 1.94	55.40 ± 1.67
n-3	6.03 ± 0.33	6.18 ± 2.70	9.84 ± 0.72	8.24 ± 2.19	9.56 ± 1.22
n-3/n-6	0.12 ± 0.01	0.12 ± 0.05	0.18 ± 0.01	0.15 ± 0.04	0.17 ± 0.03
Δ6-des index (n-6)	0.27 ± 0.01	0.33 ± 0.03	0.34 ± 0.02	0.34 ± 0.01	0.33 ± 0.01
Δ6-des index(n-3)	0.13 ± 0.03	0.26 ± 0.02	0.29 ± 0.02	0.29 ± 0.01	0.26 ± 0.02
DBI	1.45 ± 0.02	1.49 ± 0.08	1.69 ± 0.04	1.61 ± 0.13	1.67 ± 0.04
